# A physiological significance of the functional interaction between Mus81 and Rad27 in homologous recombination repair

**DOI:** 10.1093/nar/gkv025

**Published:** 2015-01-27

**Authors:** Huong Phung Thi Thu, Tuan Anh Nguyen, Palinda Ruvan Munashingha, Buki Kwon, Quy Dao Van, Yeon-Soo Seo

**Affiliations:** Department of Biological Sciences, Korea Advanced Institute of Science and Technology, Daejeon 305-701, Korea

## Abstract

Fen1 and Mus81–Mms4 are endonucleases involved in the processing of various DNA structural intermediates, and they were shown to have genetic and functional interactions with each other. Here, we show the *in vivo* significance of the interactions between Mus81 and Rad27 (yeast Fen1). The N-terminal 120 amino-acid (aa) region of Mus81, although entirely dispensable for its catalytic activity, was essential for the abilities of Mus81 to bind to and be stimulated by Rad27. In the absence of *SGS1*, the *mus81*_Δ120N_ mutation lacking the N-terminal 120 aa region exhibited synthetic lethality, and the lethality was rescued by deletion of *RAD52*, a key homologous recombination mediator. These findings, together with the fact that Sgs1 constitutes a redundant pathway with Mus81–Mms4, indicate that the N-terminus-mediated interaction of Mus81 with Rad27 is physiologically important in resolving toxic recombination intermediates. Mutagenic analyses of the N-terminal region identified two distinct motifs, named N21–26 (aa from 21–26) and N108–114 (aa from 108–114) important for the *in vitro* and *in vivo* functions of Mus81. Our findings indicate that the N-terminal region of Mus81 acts as a landing pad to interact with Rad27 and that Mus81 and Rad27 work conjointly for efficient removal of various aberrant DNA structures.

## INTRODUCTION

DNA genomes of all organisms are duplicated only once per cell cycle to produce genetically identical offspring cells. This process is faithfully executed and precisely regulated to preserve the integrity of genetic information. During DNA replication, there are a variety of barriers that can interfere with the normal progression of replication forks (RFs), including, for example, DNA lesions, aberrant structures or topological stress within the template DNA and tightly bound proteins (1–3). The failure to repair stalled or broken RFs triggers chromosome rearrangements and, thus, genome instability, leading to pathological cellular conditions or even cell death (4). Therefore, cells are equipped with a number of other pathways that cooperate with the replication machinery to avoid the hazardous outcome of DNA replication accidents. One pathway involved in the recovery of the impaired RFs is homologous recombination (HR) which entails the formation of DNA-branched intermediates that physically joint sister chromatids (3,5). Accumulation of these intermediates is potentially deleterious to cells and must be resolved through either the dissolution by helicases (for example, Sgs1) or the endonucleolytic cleavage by structure-selective endonucleases (for example, Mus81) (6–10).

Mus81 is a member of the conserved XPF family of endonucleases and is active as a heterodimer complex with a non-catalytic partner which is Eme1 in humans and fission yeasts and Mms4 in budding yeasts (11–13). In mitotic cells, the Mus81 heterodimeric complex (hereinafter referred to as Mus81 complex) catalyzes the resolution of replication- and recombination-associated DNA structures formed during repair of stalled/collapsed RFs or double-strand breaks (DSBs) (3,7,9,14–17). Biochemically, it was shown that Mus81 complex was able to cleave various DNA structures that include nicked Holliday junctions (HJs), D-loop and 3′-flap (3′F) (11–12,17–19). A Mus81 complex prefers the presence of a three- or four-way junction containing a 5′-end at the junction, which guides the incision cleavage of the complex (11,12,20–22). Genetically, it was shown that *mus81* mutants were hypersensitive to a variety of exogenous DNA damaging agents such as methyl methanesulfonate (MMS), camptothecin (CPT), hydroxyurea (HU) and ultraviolet radiation (14,15,23–25). It was also shown that *in vivo MUS81* acted in parallel or as a redundant pathway with *SGS1*, a member of the ubiquitous RecQ family of DNA helicases, to process toxic recombination intermediates (9,11,12,17–20,22,26).

Fen1 is a member of the XPG/RAD2 nuclease family and possesses 5′-flap (5′F) structure-specific endonuclease and 5′-3′ exonuclease activities, playing multiple roles in DNA replication and repair (27,28,29,30,31,32). During lagging strand DNA synthesis, Fen1 removes the 5′F structure generated by DNA polymerase δ-catalyzed strand displacement synthesis (33), producing a nick that is ligated by DNA ligase 1 (30,33–35). Besides, Fen1 is involved in the long-patch base excision repair by cleaving within the apurinic/apyrimidinic site-terminated flap (36–38). In yeasts, a null mutant of Rad27 (yeast Fen1) exhibited severe growth defects, sensitivity to DNA damaging agents, genome instability and hyper-recombination (32,39), highlighting the importance of this enzyme in many DNA transactions. In mice, deletion of both copies of *FEN1* genes led to embryonic lethality (40).

Recently, it was reported that the Mus81–Mms4 complex physically and functionally interacted with Rad27; mutations in *MUS81* and *RAD27* were synthetically lethal (41) and the two nucleases, Mus81–Mms4 and Rad27, stimulated each other's nuclease activities (42). These findings imply that the two endonucleases collaborate *in vivo* to process some structural intermediates arising during lagging strand DNA synthesis and other DNA transactions such as HR and DSB repair (42). The mutual stimulation of catalytic activities observed between Rad27 and Mus81–Mms4 was mediated by specific protein–protein interactions (42), raising the possibility that the two endonucleases should work in close proximity to efficiently remove branched single-stranded (ss) DNA structures. Many important roles of these two enzymes in DNA metabolisms prompted us to investigate the physiological significance of interactions *in vivo* and *in vitro* observed between Mus81 and Rad27 in order to delineate the cellular process that depends on their functional interaction.

In this study, we attempted to map the region of Mus81 required for its physical and functional interaction with Rad27 and analyzed the *in vivo* defects using various mutant alleles defective in their physical and functional interactions. We found that the N-terminus of Mus81 was required for Rad27 binding and that this binding was essential *in vivo* since the mutant cells impaired in this regard resulted in cell death. Therefore, our results indicate that a joint action of Mus81 and Rad27 is critical for resolving various aberrant DNA structures and toxic recombination intermediates to repair DNA replication errors and other DNA damages.

## MATERIALS AND METHODS

### Yeast strains

NJY1777 (*MAT*a*ade2–1 ade3::hisG ura3–1 his3–11,15 trp1–1 leu2–3,112 lys2 mus81–10::KAN sgs1–20::hphMX4 can1–100 +* pJM500*-URA3-SGS1*) and MIY2343 (*MAT*a *ade2–1 ade3::hisG ura3–1 his3–11,15 trp1–1 leu2–3,112 lys2 rad52Δ::TRP1 mus81–10::KAN sgs1–20::hphMX4 can1–100* + pMI6337-*URA3-mus81–2*) were kind gifts from Dr Miki Ii at University of Alaska Anchorage (AK, USA) (43). HY1728 (*MAT*a*ade2-1 trp1-1 leu2-3,112 his3-11–15 ura3 can1-100 mms4Δ::HPHMX4*) was a courtesy from Dr Dana Branzei at IFOM-IEO (Milan, Italy) (44).

### Enzymes, antibodies, DNA and nucleotides

Restriction endonucleases, DNA polymerases and polynucleotide kinase were purchased from Enzynomics (Daejeon, Korea). Antibodies against hexahistidine (6XHis) epitope or glutathione-S transferase (GST) used for western blotting were from Qiagen (Valencia, CA, USA) and Santa Cruz Biotechnology (Santa Cruz, CA, USA), respectively. Secondary antibodies were from Amersham Biosciences (Piscataway, NJ, USA). Oligonucleotides used in this study were commercially synthesized by Genotech or Macrogen (Daejeon, Korea). All oligonucleotides were gel-purified prior to use. Nucleoside triphosphates were obtained from Sigma-Aldrich (St. Loius, MO, USA), and [γ-^32^P]ATP (>3000 Ci/mmol) was purchased from Perkin Elmer NEN (Waltham, MA, USA). The pRS plasmids were purchased from New England Biolabs (Beverly, MA, USA). The pET vectors used for protein expression in *Escherichia coli* were from Novagen (Darmstadt, Germany). Isopropyl β-D-1-thiogalactopyranoside (IPTG) was from ElpisBiotech (Daejeon, Korea). Imidazole (IDZ) was from Acros Organics (Geel, Belgium). The uracil analog, 5-fluoroorotic acid (5-FOA) and proteinase K were obtained from Duchefa Biochemie (Haarlem, Netherland). MMS was obtained from Sigma-Aldrich (St. Loius, MO, USA).

### Preparation of DNA substrates

The oligonucleotides used to construct DNA substrates are listed in Table 1. The preparation of DNA substrates and their labeling at the 5′ end were carried out as described previously (45). To make the 3′F substrate, oligonucleotides 1, 3 and 5 in Table 1 were used. To make the 5′-double-flap substrate (5′DF), oligonucleotides 1, 2 and 4 were used. Locations of radioisotopic labels in substrates are indicated by asterisks in each figure. All substrates were purified by polyacrylamide gel electrophoresis (PAGE) prior to use as described (45).

**Table 1.** Oligonucleotides used to construct DNA substrates in this study

No.	Nucleotide sequences (length in nt)	Name
1	5′-CGAACAATTCAGCGGCTTTAACCGGACGCTCGACGCCATTAATAATGTTTTC-3′ (52)	729
2	5′-GAAAACATTATTAATGGCGTCGAGCTAGGCACAAGGCGAACTGCTAACGG-3′ (50)	5TY-1
3	5′-CCGTTAGCAGTTCGCCTTGTGCCTAACTGCATACGAACTGATAGGATGCG-3′ (52)	Anti729-1
4	5′-CCGTTAGCAGTTCGCCTTGTGCCTAG-3′ (26)	5TBG
5	5′-ACTGCATACGAACTGATAGGATGCG-3′ (25)	Anti729-2

### GST pull-down assays

Crude extracts (10 pmol equivalent with respect to purified Rad27 and Mus81 proteins) from *E. coli* cells expressing Rad27 (with a GST tag fused to its N-terminus) or full-length Mus81/its truncated versions (with a 6XHis tag fused to its C-terminus) were mixed and incubated with 0.2 ml of glutathione-agarose beads (Amersham Biosciences) in binding buffer (25-mM Tris-HCl/pH 8.0, 100-mM NaCl, 10% glycerol, 0.1% NP-40). After rocking at 4°C for 2 h, the beads were collected by centrifugation, washed three times with 1 ml of binding buffer and the bound proteins were subjected to 10% sodium dodecyl sulphate (SDS)-PAGE, followed by western blotting using anti-6XHis or anti-GST antibodies.

### Protein purification

In order to prepare Mus81–Mms4_Δ40N_, we co-transformed two plasmids, pET28a expressing Mus81 with a 6XHis fused to its C-terminus and pET21d expressing Mms4_Δ40N_, a truncated version of Mms4 devoid of the N-terminal 40 aa residues into *E. coli* BL21-CodonPlus (DE3)-RIL strain (Strategene; La Jolla, CA, USA). Cells (2 l) were grown at 37°C to OD_600_ = 0.5–0.7 and expression of proteins was induced with 0.5-mM IPTG, followed by 4-h incubation at 25°C. Cells were then harvested by centrifugation, and the resulting cell pellet was resuspended in 100 ml of lysis buffer T_100_ (50-mM Tris-HCl/pH 8.0, 100-mM NaCl, 10% glycerol, 0.2% NP-40 and protease inhibitors). The subscript number in T_100_ indicates the concentration of NaCl in mM. Following sonication, the crude lysate was clarified by centrifugation at 45 000 rpm in a Beckman 70Ti rotor for 20 min. The supernatant (95 ml, 5.2 mg/ml) was directly loaded onto a phosphocellulose column (15 ml, Φ1.5 × 8.5 cm) pre-equilibrated with buffer T_100_. After extensive washing with buffer T_100_, the column was eluted stepwise with buffer T_200_ and buffer T_500_. The peak protein fractions from T_500_ elution were pooled (40 ml, 7 mg/ml) and loaded directly onto a Ni^2+^-NTA column (1 ml, Φ 0.7 × 2.5 cm) equilibrated with buffer T_500_. After extensive washing with buffer T_500_ plus 50-mM IDZ, the bound proteins were eluted with buffer T_500_ plus 500-mM IDZ. Next, an aliquot (200 μl, 0.17 mg/ml) of the peak fraction was subjected onto glycerol gradient sedimentation (5 ml, 15–35% glycerol in buffer T_500_) at 45 000 rpm for 24 h in a Beckman SW55Ti rotor. Glycerol gradient fractions obtained were then stored at −80°C and used for all subsequent biochemical assays. The wild-type Mus81–Mms4 complex used as positive control was expressed using pET28-His_6_-*MMS4*-*MUS81* (42) and was also purified as described above for Mus81–Mms4_Δ40N_. The other derivatives of Mus81 complex such as Mus81_Δ120N_–Mms4_Δ40N_, Mus81_Δ21–22_–Mms4_Δ40N_, Mus81_Δ21–24_–Mms4_Δ40N_ and Mus81_Δ21–26_–Mms4_Δ40N_ (see below in the Results section) were also purified with the same procedure as describe above.

The plasmid pNJ6125 expressing Slx1–Slx4, a kind gift from Dr Steven J. Brill at Rutgers University (NJ, USA), was transformed into *E. coli* BL21-CodonPlus (DE3)-RIL strain (46). The catalytic subunit Slx4 possesses a 6XHis epitope fused to its N-terminus. Cleared lysate from cells (1 l) was prepared similarly as described above for the Mus81 complexes. The supernatant (50 ml) was mixed with an SP-sepharose (10 ml) and incubated with gentle rocking for 2 h at 4°C for batch absorption. The mixture was then poured into a column (Φ 1.5 × 5.5 cm) and then washed extensively with buffer T_100_, followed by elution with a linear gradient (100 ml total) from T_100_ to T_1000_. The fractions containing Slx1–Slx4 were confirmed by western blotting using anti-6XHis antibodies, and the peak fractions were pooled. The pooled fractions (15 ml, 1.5 mg/ml) were incubated with 1 ml of Ni^2+^-NTA resin for batch absorption for 2 h at 4°C with gentle rocking. The mixture was then poured into a column (Φ 0.7 × 2.5 cm). After two successive washing with buffer T_500_ plus 25 mM and T_500_ plus 50-mM IDZ (10 ml each), the bound proteins were eluted with buffer T_500_ plus 500-mM IDZ. Next, an aliquot (200 μl, 0.21 mg/ml) of the peak fraction was subjected onto glycerol gradient sedimentation as described above.

### Nuclease assays

Reaction mixtures and conditions to measure enzymatic activities of Mus81–Mms4 complexes and Rad27 were as described previously (42) with amounts of enzymes and DNA substrates indicated in relevant figures.

### Drop dilution assays

The plasmids pRS325 containing wild-type or mutant alleles of Mus81, expression of which is driven by ADH1 promoter, were transformed into NJY1777. The transformants were first grown on plates, and single colonies formed were inoculated into liquid media (1 ml) until saturation. Cell densities were adjusted to OD_600_ = 1 (∼2 x 10^7^ cells/ml) by diluting with dH_2_O, followed by spotting of 10-fold serial dilutions onto plates with or without DNA damaging agents. The plates were then incubated at 30°C for 3–4 days. Suppression of synthetic lethality of *sgs1Δmus81Δ* double null mutants by a variety of *mus81* mutant alleles constructed was examined in the presence of 5-FOA. To test the drug sensitivity of *mus81* mutant alleles in the *sgs1Δ* null strain, cells were spotted on plates containing different concentrations of MMS or other agents as indicated in each relevant figure.

## RESULTS

### The N-terminal 120 aa region of Mus81 is required for binding Rad27

**Figure 1.**
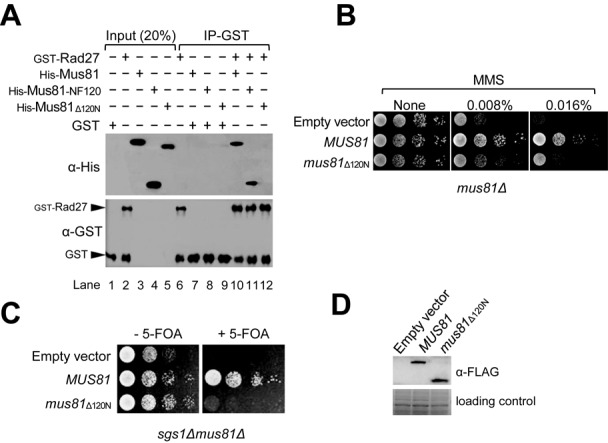
The N-terminal region of Mus81 consisting of 120 aa residues is responsible for binding Rad27. (**A**) Crude extracts containing GST-Rad27 (10 pmol) and Mus81–6XHis derivatives (10 pmol each; Mus81, the full-length Mus81 subunit; Mus81N120, the N-terminal 120 aa fragment; Mus81_Δ120N_, Mus81 lacking the N-terminal 120 aa residues) were mixed and incubated with glutathione-agarose beads for 2 h at 4°C with gentle rocking. The GST-Rad27/Mus81 complex formed was collected and washed. The presence of Mus81 derivatives and Rad27 in the precipitated materials was determined by western blotting using anti-6XHis (α-His) and anti-GST (α-GST) polyclonal antibodies, respectively. Input (20%) means the amount of proteins loaded for western blotting was 20% of total proteins used for the pull-down assays. IP-GST: GST immunoprecipitation. (**B**) The MMS sensitivity of *mus81*_Δ120N_ was examined. The NJY1777 (*sgs1Δmus81Δ* + pJM500*-URA3-SGS1*) strain was transformed with an empty vector or vectors containing *MUS81* or *mus81*_Δ120N_ driven by an ADH1 promoter. The transformants were grown until saturation in liquid media and then spotted onto plates without or with indicated amounts of MMS. The plates were incubated at 30°C for 4 days. (**C**) The complementation of *sgs1Δmus81Δ* synthetic lethality by *mus81* mutant alleles. The transformants (in panel (B)) were grown until saturation in liquid media and then spotted onto plates with or without 5-FOA. (**D**) Analysis of protein expression in 10% SDS-PAGE. The gels were stained with Coomassie blue for the loading control (bottom part). The expression of Mus81 and Mus81_Δ120N_ was confirmed by western blotting using anti-FLAG (α-FLAG) monoclonal antibodies.

As an attempt to understand the *in vivo* function of the genetic and physical interactions between Mus81 and Rad27, we decided to map the domain of Mus81 required to bind Rad27. Considering that the C-terminal part of Mus81 is necessary and sufficient to form a complex with Mms4 (24) and that binding of bulky proteins to the same protein could be mutually exclusive, the N-terminal part of Mus81 is most likely responsible to bind another interacting partner, namely, Rad27. Therefore, we first constructed expression vectors that produced a series of C-terminally truncated Mus81 fragments, i.e. Mus81-NF474, Mus81-NF316 and Mus81-NF158 (NF denotes N-terminal Fragment; the number indicates aa residues from the N-terminus of Mus81). Using the GST pull-down assay as described in the Materials and Methods section, we examined which one of the Mus81 truncated fragments was able to bind Rad27. The result was that all three fragments tested were able to bind Rad27 with efficiencies nearly comparable to that of full-length Mus81 (Supplementary Figure S1). This is in keeping with our prediction above and indicates that the domain responsible for binding Rad27 resides within the N-terminal 158 aa region of Mus81. Next, we further truncated Mus81-NF158 into two smaller fragments, Mus81-NF120 and Mus81-NF80. We found that Mus81-NF120 (the N-terminal 120 aa fragment of Mus81) bound Rad27 as efficiently as the full-length Mus81 subunit when we used the GST pull-down assays (Figure 1A, compare lanes 10 and 11), but Mus81-NF80 failed to do so (Supplementary Figure S2A). In addition, Mus81_Δ120N_ (the mutant Mus81 subunit devoid of the N-terminal 120 aa residues) failed to bind Rad27 (Figure 1A, lane 12).

In order to define *in vivo* defects associated with the inability of Mus81 to physically interact with Rad27, we examined MMS sensitivity of *mus81*_Δ120N_ mutant cells through drop dilution assays. As expected, the *mus81Δ* null cells (Figure 1B, empty vector) were highly sensitive to MMS, growing poorly in the presence of 0.008% MMS in contrast to wild-type cells (Figure 1B, *MUS81*). The *mus81*_Δ120N_ cells were also highly sensitive to MMS (Figure 1B, *mus81*_Δ120N_), although slightly less than *mus81Δ* null mutants, implying that *mus81*_Δ120N_ is defective in overcoming or repairing MMS-induced DNA damages. It is known that a cellular process such as DNA damage repair often leads to the formation of joint-molecule intermediates via HR. These intermediates can be resolved by two redundant pathways; one requires Sgs1–Top3–Rmi1, and the other Mus81–Mms4. The function of Mus81 becomes essential when Sgs1 is not available or vice versa (9,11,12,17–20,22,26). In order to further delineate defective cellular processes associated with the N-terminal 120 aa deletion of Mus81 in this regard, we used *sgs1Δmus81Δ* double mutant cells that were rendered viable with a plasmid expressing wild-type Sgs1. With the control empty vector transformed, *sgs1Δmus81Δ* cells became unviable after they had lost the plasmid expressing Sgs1 (Figure 1C, empty vector). As expected, they grew well in the presence of episomal copies of wild-type *MUS81* (Figure 1C, *MUS81*). In contrast, we found that the presence of *mus81*_Δ120N_ allele failed to support the growth of *sgs1Δmus81Δ* cells (Figure 1C, *mus81*_Δ120N_). As shown in Figure 1D, expression levels of wild-type Mus81 and mutant Mus81_Δ120N_ proteins were comparable, excluding the possibility that the marked phenotypic difference was due to differences in expression levels. These findings above suggest that the *in vivo* defects caused by the loss of the N-terminal 120 aa region are most likely attributable to the loss of interactions of Mus81 with Rad27. Our results raise strongly the possibility that Rad27 via its physical interaction with Mus81 could contribute significantly to HR repair (HRR) pathways together with Sgs1.

### The deletion of the N-terminal region did not affect enzymatic activity of Mus81 complex

**Figure 2.**
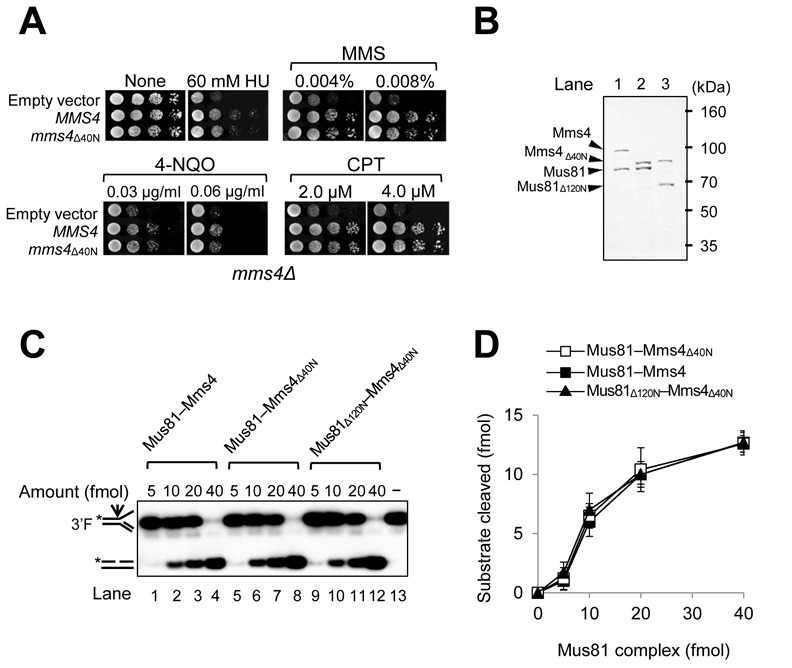
The N-terminal 120 aa region of Mus81 is dispensable for the catalytic endonuclease activities of Mus81 complexes. (**A**) Complementation activity of *mms4*_Δ40N_ to rescue the drug sensitivity of *mms4Δ* strain. *MMS4* or *mms4*_Δ40N_ under the control of its native promoter in the pRS314 vector was transformed into HY1728 strain (*mms4Δ*). The transformants were grown until saturation in liquid media and then spotted onto plates with indicated amounts of DNA damaging agents. The plates were incubated at 30°C for 3 days. HU, hydroxyurea; MMS, methyl methanesulfonate; 4-NQO, 4-nitroquinoline oxide; CPT, camptothecin. (**B**) Purification of recombinant Mus81–Mms4, Mus81–Mms4_Δ40N_ and Mus81_Δ120N_–Mms4_Δ40N_ complexes. SDS-PAGE (10%) analysis of purified Mus81 complexes, followed by Coomassie blue staining. The sizes of molecular mass marker are indicated in kDa. (**C**) Comparison of endonuclease activities of three recombinant Mus81 complexes from panel (B). Reactions were carried out in standard reaction mixtures containing 15 fmol of 3′F substrate and increasing amounts (5, 10, 20 and 40 fmol) of Mus81 complexes. Reactions were incubated at 30°C for 30 min and terminated by the addition of 0.2% SDS, 10-μg proteinase K, followed by incubation at 37°C for 15 min. The products were subjected to a 10% PAGE in 0.5X TBE at 150 V and the gels were dried and autoradiographed. The structures of 3′F substrate and the cleavage product are as illustrated in the figure. The arrow on the substrate denotes the site of cleavage. Asterisks indicate the position of ^32^P-label at the 5′ DNA ends. (**D**) The amount (fmol) of cleavage products formed by the endonuclease activity of Mus81 complexes on 3′F substrate (in panel (C)) was plotted against the amount (fmol) of Mus81 complexes used. The graph with error bars indicated was obtained from four independent experiments. The error bars represent the standard deviation from the mean of four independent experiments.

It was reported that the N-terminal 48 aa residues of Mms4 were dispensable for its abilities to form a complex with Mus81 and to complement the *mms4Δ* null mutation (24). We found that the co-expression of the N-terminal 40 and 80 aa truncated Mms4 (Mms4_Δ40N_ and Mms4_Δ80N_, respectively) with Mus81 in *E. coli* led to increase in the overall yield of Mus81 complexes (data not shown). Prior to use of Mms4_Δ40N_ in subsequent preparation of various Mus81 complexes, we decided to investigate whether the *mms4*_Δ40N_ mutation is defective in repair of various DNA damages induced by HU, MMS, 4-nitroquinoline 1-oxide (4-NQO) and CPT. As shown in Figure 2A, we observed that *mms4*_Δ40N_ was able to complement the sensitivity of *mms4Δ* to all DNA damaging agents tested as efficiently as wild-type *MMS4* (Figure 2A). Based on these findings, we decided to use Mms4_Δ40N_ in place of Mms4 and purified Mus81–Mms4_Δ40N_ and Mus81_Δ120N_–Mms4_Δ40N_ (Figure 2B, lanes 2 and 3). In addition, we also purified wild-type Mus81–Mms4 to compare specific activities (Figure 2B, lane 1) using the procedure as described in the Materials and Methods section. When we compared the endonuclease activities between Mus81–Mms4_Δ40N_ and Mus81_Δ120N_–Mms4_Δ40N_ with respect to the wild-type complex, we discovered that all three complexes displayed nearly identical endonuclease activities with 3′F substrate. Thus, the N-terminal 40 aa deletion of Mms4 did not affect the endonuclease activity of Mus81 complex (Figure 2C, compare lanes 1–4 and 5–8; Figure 2D), and Mus81_Δ120N_–Mms4_Δ40N_ exhibited the level of endonuclease activity comparable to that of Mus81–Mms4_Δ40N_ (Figure 2C, compare lanes 5–8 and 9–12; Figure 2D). We also investigated the *in vivo* levels of Mus81_Δ120N_–Mms4 complex and the endonuclease activity of the mutant complex in comparison with wild type. As shown in Supplementary Figure S3, the amount of Mus81_Δ120N_-Mms4 immunoprecipitated with anti-FLAG antibodies was similar to that of wild-type complex when we used the same number of cells to prepare extracts for immunoprecipitation (Supplementary Figure S3A). We also measured the endonuclease activities using the immunoprecipitated materials as sources of enzymes with the 3′F substrate and found that both exhibited comparable enzymatic activities (Supplementary Figure S3B). These results indicate that the deletion of N-terminus of Mus81 did not affect the Mus81-Mms4 heterodimer assembly *in vivo* and its specific endonuclease activity. Taken together, these results confirm that the loss of catalytic activity of Mus81_Δ120N_ was not the underlying cause of the synthetic lethality of *sgs1Δmus81*_Δ120N_.

**Figure 3.**
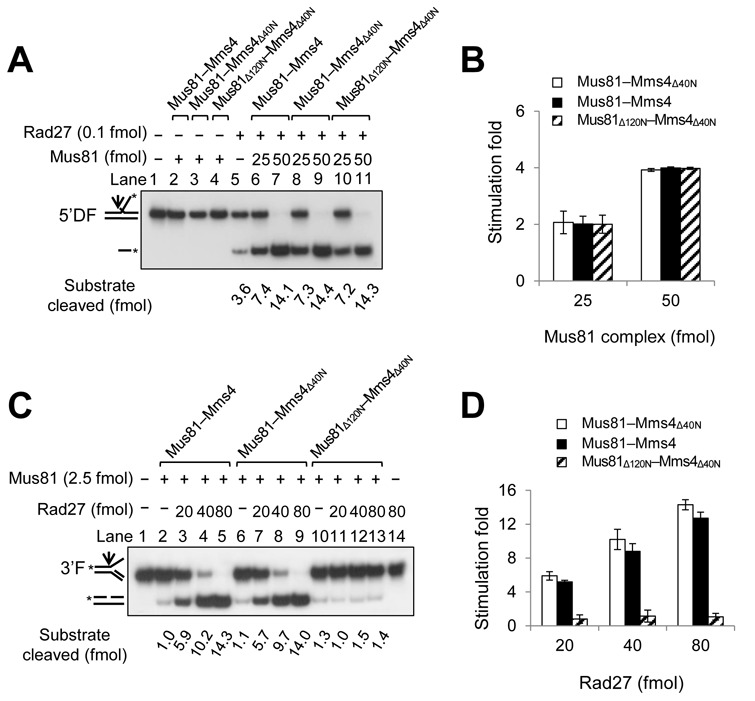
The N-terminal 120 aa region of Mus81 is required for Rad27-mediated stimulation of nuclease activity of Mus81 complex. (**A**) The Mus81_Δ120N_ mutant complex stimulated Rad27 activity as efficiently as wild-type Mus81 complex. Reactions were carried out in standard reaction mixtures containing 15 fmol of 5′DF substrate with 0.1 fmol of Rad27 and increasing amounts (25 and 50 fmol) of three different Mus81 complexes (Mus81–Mms4, Mus81–Mms4_Δ40N_ and Mus81_Δ120N_ –Mms4 _Δ40N_). Reactions were incubated for 30 min at 37°C, followed by the addition of 4 μl of 6X stop solution (40% sucrose, 60-mM EDTA, 1.2% SDS, 0.05% bromophenol blue and 0.05% xylene cyanol). The cleavage products were subjected to electrophoresis and the gels were dried and autoradiographed. (**B**) **T**he amount (fmol) of cleavage products formed by the endonuclease activity of Rad27 on 5′DF substrate (in panel (A)) was plotted against the amount (fmol) of Mus81 complexes added. The graph with error bars indicated was obtained from four independent experiments. The error bars represent the standard deviation from the mean of four independent experiments. (**C**) Rad27 was not able to stimulate the endonuclease activity of Mus81 complex lacking the N-terminal 120 aa region. Reactions were carried out in standard reaction mixtures containing 15 fmol of 3′F substrate with 2.5 fmol of each of the three different Mus81 complexes and increasing amounts (20, 40 and 80 fmol) of Rad27. Reactions were incubated at 30°C for 30 min and terminated. The products were subjected to a 10% PAGE in 0.5X TBE at 150 V, and the gels were dried and autoradiographed. (**D**) The amount (fmol) of cleavage products formed from 3′F substrate (in panel (C)) by Mus81 complexes was plotted against the amount (fmol) of Rad27 added. The graph with error bars was obtained from four independent experiments. The error bars represent the standard deviation from the mean of four independent experiments.

Next, we examined whether the N-terminal 120 aa deletion of Mus81 could lead to the impairment of its ability to stimulate the endonuclease activity of Rad27 or vice versa. We found that all three Mus81 complexes, Mus81–Mms4, Mus81–Mms4_Δ40N_ and Mus81_Δ120N_–Mms4_Δ40N_, stimulated Rad27 activity to similar extents as shown in Figure 3A and B. When we used increasing levels (0, 25, 50 fmol) of Mus81 complexes in the presence of a fixed amount (0.1 fmol) of Rad27, Rad27-catalyazed cleavage of 5′DF increased ∼2–4 fold. Both Mus81–Mms4_Δ40N_ (Figure 3A, lanes 8 and 9) and Mus81_Δ120N_–Mms4_Δ40N_ (lanes 10 and 11) stimulated Rad27 activity as efficiently as wild-type Mus81–Mms4 (lanes 6 and 7). This indicates that the N-terminal regions of both Mus81 and Mms4 are not essential to stimulate the endonuclease activity of Rad27. However, we found that Rad27 enhanced the endonuclease activities of Mus81–Mms4 and Mus81–Mms4_Δ40N_ to similar extents (Figure 3C, compare lanes 2–5 and 6–9; Figure 3D), but failed to do so when Mus81_Δ120N_–Mms4_Δ40N_ was used (Figure 3C, lanes 10–13; Figure 3D). The results above suggest that the N-terminal 120 aa region of Mus81 is important not only for binding Rad27 but also for the Rad27-mediated stimulation of Mus81 activity.

### The two separate motifs in the N-terminus of Mus81 are critical for its functional and physical interaction with Rad27

**Figure 4.**
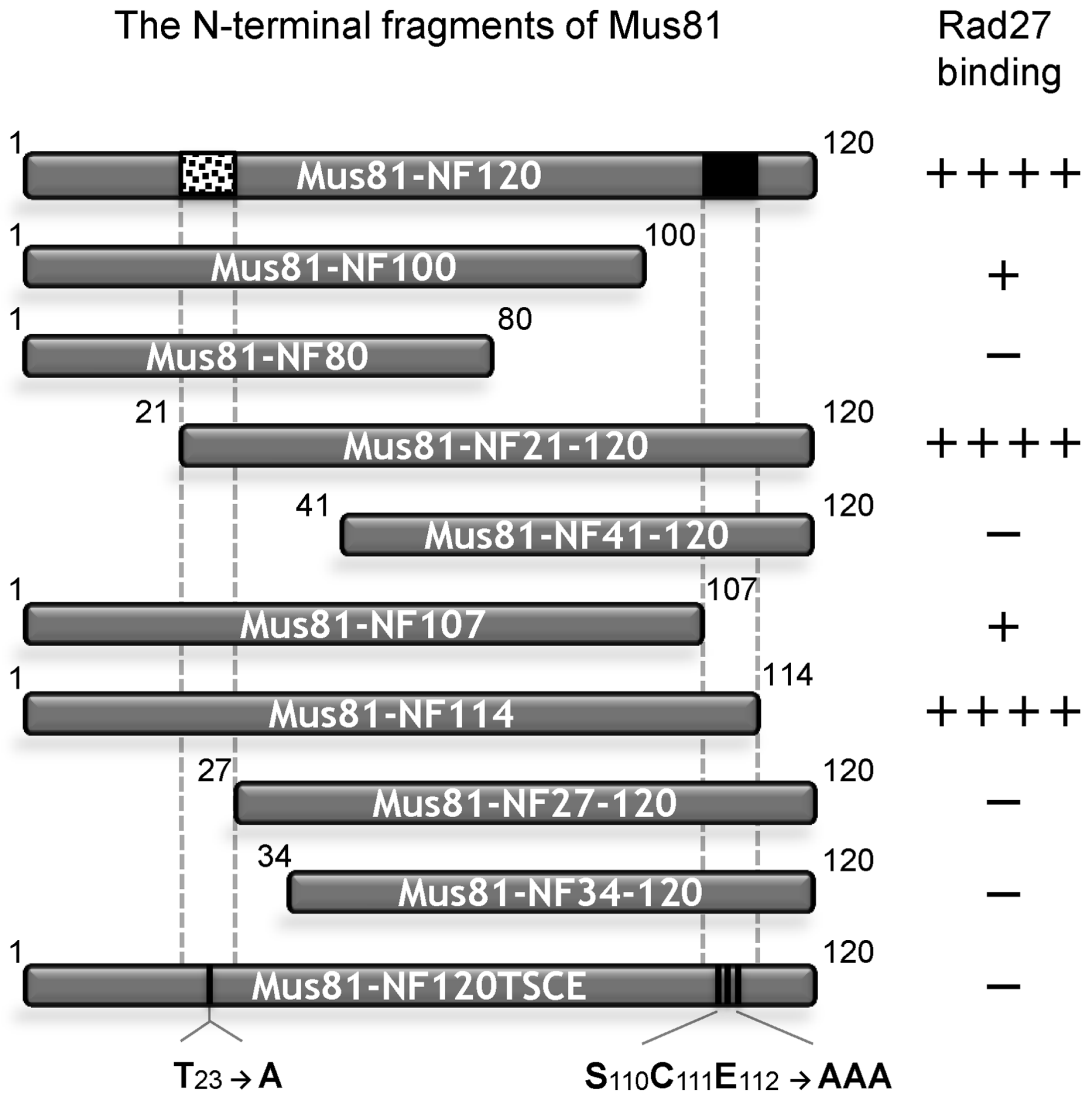
Mapping of the specific residues at the N-terminus of Mus81 required for interaction with Rad27. The schematic summary of results from GST pull-down assays with a series of derivatives of the N-terminal 120 aa fragment of Mus81 and GST-Rad27. The numbers in each fragment indicate the positions of aa in full-length Mus81. The N21–26 and N108–114 motifs are indicated by dotted and filled boxes in the Mus81-NF120, respectively. Mus81-NF120TSCE containing aa substitution (indicated by vertical lines) is also shown.

Our findings above that the cellular defect of *mus81*_Δ120N_ in *sgs1Δ* null cells was directly associated with the loss of interaction of Mus81 with Rad27 demonstrate the *in vivo* importance of the specific protein–protein interaction between the N-terminus of Mus81 and Rad27. In order to understand this specific protein–protein interaction, we decided to determine which part within the N-terminal 120 aa region of Mus81 is important to interact with Rad27. To this end, we prepared a series of truncated derivatives of Mus81-NF120 as shown in Figure 4. Using GST pull-down assays as described in the Materials and Methods section, we investigated the ability of each derivative of Mus81-NF120 to bind Rad27 *in vitro* (Supplementary Figure S2). The result was that Mus81-NF21–120 and Mus81-NF114 bound Rad27 as efficiently as the Mus81-NF120. The two fragments, Mus81-NF100 and Mus81-NF107, were able to bind Rad27 weakly. All other fragments including Mus81-NF80, Mus81-NF41–120, Mus81-NF27–120 and Mus81-NF34–120 failed to bind Rad27. Analysis of the results obtained from the GST pull-down assays using all the derivatives revealed that the two small motifs were important for Rad27 binding activity: one (N21–26) from aa 21–26 and the other (N108–114) from aa 108–114.

### Analysis of the roles of the two motifs in proper functioning of Mus81 *in vivo*

**Figure 5.**
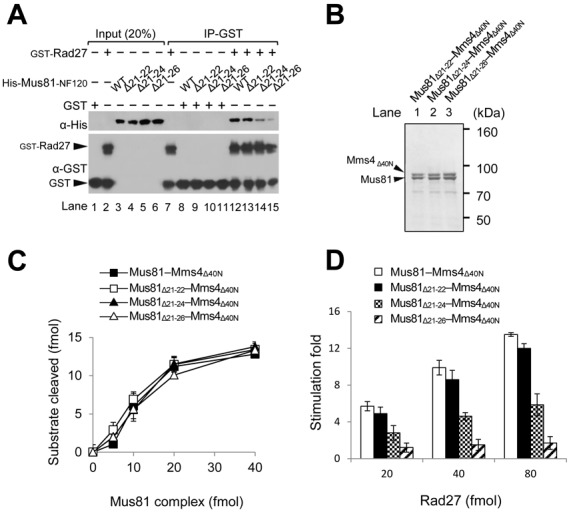
The importance of the N21–26 motif for the interaction of Mus81-NF120 with Rad27. (**A**) GST pull-down assays with GST-Rad27 and derivatives of Mus81-NF120 (WT) that include Mus81-NF120Δ21–22 (Δ21–22), Mus81-NF120Δ21–24 (Δ21–24) and Mus81-NF120Δ21–26 (Δ21–26) (see text for details). (**B**) SDS-PAGE (8%) analysis of purified recombinant Mus81_Δ21–22_–Mms4_Δ40N_, Mus81_Δ21–24_–Mms4_Δ40N_ and Mus81_Δ21–26_–Mms4_Δ40N_ complexes. The gel was Coomassie-blue stained. The sizes of molecular mass marker are indicated in kDa. (**C**) Comparison of endonuclease activities of three recombinant Mus81_Δ21–22_–Mms4_Δ40N_, Mus81_Δ21–24_–Mms4_Δ40N_ and Mus81_Δ21–26_–Mms4_Δ40N_ complexes. Reactions were carried out in standard reaction mixtures containing 15 fmol of 3′F substrate and increasing amounts (5, 10, 20 and 40 fmol) of Mus81 complexes. The amount (fmol) of cleavage products formed by the endonuclease activity of Mus81 complexes on 3′F substrate was plotted against the amount (fmol) of Mus81 complexes added. The graph with error bars indicated was obtained from four independent experiments. The error bars represent the standard deviation from the mean of four independent experiments. (**D**) The influence of Rad27 on the endonuclease activities of Mus81_Δ21–22_–Mms4_Δ40N_, Mus81_Δ21–24_–Mms4_Δ40N_ and Mus81_Δ21–26_–Mms4_Δ40N_ complexes. Reactions were carried out and the graph obtained was presented as described in Figure 3C.

To further narrow down the N21–26 motif, we prepared constructs that expressed a series of deletion derivatives of the Mus81-NF120, which include Mus81-NF120_Δ21–22_, Mus81-NF120_Δ21–24_ and Mus81-NF120_Δ21–26_ lacking two, four and six aa residues, respectively, from aa position 21. With the crude extracts containing one of these derivatives, we then examined the ability of each derivative to interact with Rad27 using the procedure as described in the Materials and Methods section. As shown in Figure 5A, Mus81-NF120_Δ21–22_ was able to bind Rad27 as efficiently as wild-type Mus81-NF120 (Figure 5A, compare lanes 12 and 13). In contrast, Mus81-NF120_Δ21–24_ and Mus81-NF120_Δ21–26_ showed significantly reduced or poor, respectively, Rad27-binding activity (Figure 5A, compare lanes 12–13 and 14–15). We also purified Mus81_Δ21–22_–Mms4_Δ40N_, Mus81_Δ21–24_–Mms4_Δ40N_ and Mus81_Δ21–26_–Mms4_Δ40N_ to near homogeneity (Figure 5B) and compared their specific activities with 3′F substrate. The result is that all three Mus81 mutant proteins gave rise to the wild-type (Mus81–Mms4_Δ40N_) level of endonuclease activity (Figure 5C), consistent with our finding above that the N-terminal 120 aa deletion of Mus81 protein did not affect the catalytic activity of the complex. As shown in Figure 5D, the extent of stimulation of Mus81_Δ21–22_–Mms4_Δ40N_ activity by Rad27 was the most robust (∼5–12-fold, comparable to that of Mus81–Mms4_Δ40N_), followed by Mus81_Δ21–24_–Mms4_Δ40N_ (∼3–6-fold). Mus81_Δ21–26_–Mms4_Δ40N_, however, was hardly stimulated by Rad27 (Figure 5D). This result is in keeping with the results from GST pull-down assays above (Figure 5A), confirming that the physical interaction between the N-terminus of Mus81 and Rad27 is essential for a functional interaction such as for Rad27-mediated enzymatic stimulation of Mus81.

**Figure 6.**
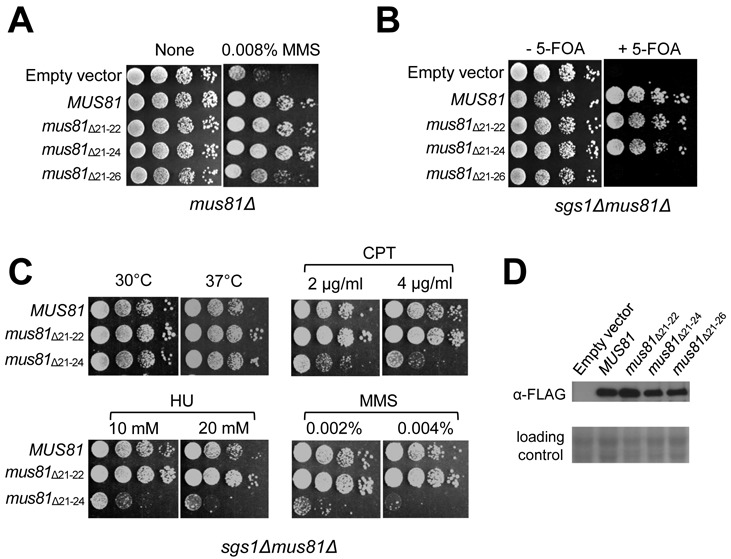
The cellular defects associated with impairment of the N21–26 motif. (**A**) The MMS sensitivity of *mus81*_Δ21–22_, *mus81*_Δ21–24_ and *mus81*_Δ21–26_ was examined. NJY1777 (*sgs1Δmus81Δ* + pJM500*-URA3-SGS1*) strain was transformed with empty vector or vectors containing *MUS81, mus81*_Δ21–22_, *mus81*_Δ21–24_ and *mus81*_Δ21–26_ driven by ADH1 promoter. The transformants were grown until saturation in liquid media and then spotted onto plates without or with different amounts of MMS concentration, which is shown on the top of each panel. The plates were incubated at 30°C for 4 days. (**B**) The complementation of *sgs1Δmus81Δ* synthetic lethality by *mus81* mutant alleles. The transformants (in panel (A)) were grown until saturation in liquid media and then spotted onto plates with or without 5-FOA. The plates were incubated at 30°C for 4 days. (**C**) The sensitivity of *sgs1Δmus81*_Δ21–22_ and *sgs1Δmus81*_Δ21–24_ to CPT, HU and MMS was examined. The colonies that grew on the 5-FOA plates from (B) were inoculated into liquid media until saturation and then spotted onto plates containing indicated amount of DNA damage agents, which are shown on the top of each panel. (**D**) Analysis of expressions of proteins in 10% SDS-PAGE. The gels were stained with Coomassie blue for loading control (bottom). The expression of Mus81 and its derivatives was confirmed by western blotting using anti-FLAG monoclonal antibodies.

In order to establish cellular defects associated with *mus81*_Δ21–22_, *mus81*_Δ21–24_ and *mus81*_Δ21–26_ mutant alleles, we decided to examine the sensitivity of each mutant allele to MMS using drop dilution assays as described in the Materials and Methods section. We found that *mus81*_Δ21–22_ and *mus81*_Δ21–24_ could fully complement both the MMS sensitivity of *mus81Δ* (Figure 6A) and the synthetic lethality of *sgs1Δmus81Δ* (Figure 6B) as efficiently as wild-type *MUS81*. In contrast, *mus81*_Δ21–26_ was able to partially restore the MMS sensitivity of *mus81Δ* (Figure 6A, *mus81*_Δ21–26_), but not the viability in the absence of a functional copy of *SGS1* (Figure 6B, *mus81*_Δ21–26_). In the absence of *SGS1, mus81*_Δ21–22_ and *mus81*_Δ21–24_ did not display any temperature-sensitive growth defect (Figure 6C, compare growth at 30 and 37°C). However, *mus81*_Δ21–24_ but not *mus81*_Δ21–22_, in the absence of *SGS1*, exhibited significant sensitivities to various DNA damaging agents such as CPT, HU and MMS (Figure 6C). These results again demonstrate that the N21–26 has a significant role in the proper functioning of Mus81 *in vivo*, especially in the absence of Sgs1. The comparable expression levels of all proteins exclude the possibility that the phenotypic differences observed with *mus81*_Δ21–22_, *mus81*_Δ21–24_ and *mus81*_Δ21–26_ mutant alleles were attributable to the differences in cellular protein levels (Figure 6D). Noteworthy is that both *mus81*_Δ120N_ and *mus81*_Δ21–26_ (with relatively a small deletion) were synthetic lethal with *sgs1Δ*, but differed in their abilities to repair MMS-induced DNA damage (compare Figures 1B and 6A). Thus, it appears that the defects of *mus81* mutant alleles in the repair of MMS-induced damages are not directly related to their synthetic lethality in combination with *sgs1Δ*.

### Determination of aa residues in the two structural motifs for stable binding of Rad27

**Figure 7.**
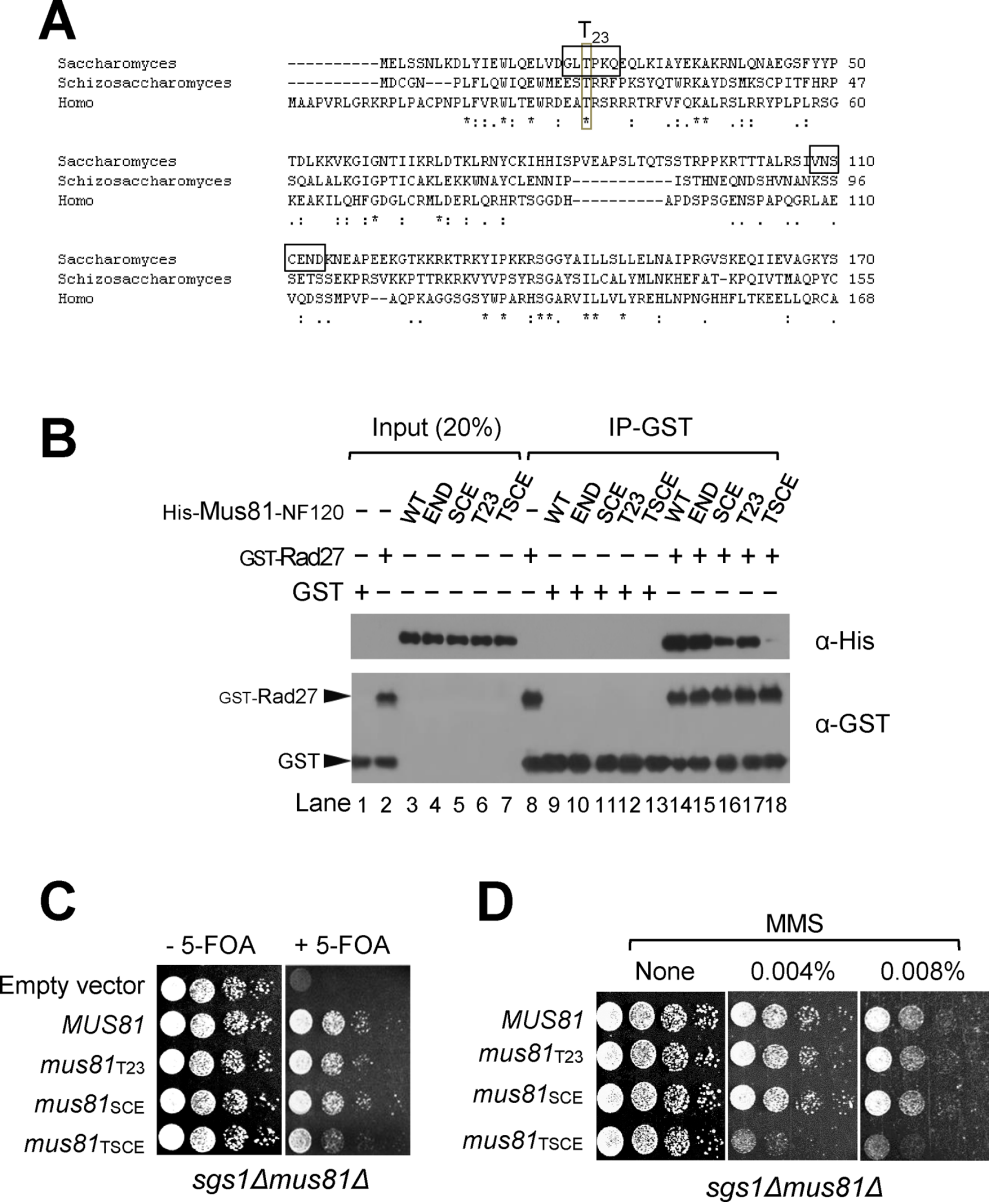
Both N21–26 and N108–114 motifs are important for the cellular function of Mus81–Mms4 complex in the absence of Sgs1. (**A**) Alignment of the N-termini of Mus81 subunits from *Saccharomyces cerevisae, S. pombe* and *H. sapiens*. The N21–26 and N108–114 motifs in yeast Mus81 N-terminus are indicated by opened boxes. (**B**) GST pull-down assays with GST-Rad27 and derivatives of Mus81-NF120 (WT) including Mus81-NF120END (END), Mus81-NF120SCE (SCE), Mus81-NF120T23 (T23) and Mus81-NF120TSCE (TSCE). All aa residues of interest (T_23_, S_110_, C_111_, E_112_, N_113_ and D_114_) were changed into alanine. The GST pull-down assays were carried out as described in the Materials and Methods section. (**C**) The complementation of *sgs1Δmus81Δ* synthetic lethality by *mus81* mutant alleles that contained either T_23_→A or S_110_C_111_E_112_→AAA or both substitutions. NJY1777 (*sgs1Δmus81Δ* + pJM500*-URA3-SGS1*) strain was transformed with an empty vector or vectors containing *MUS81* and *mus81* mutant alleles driven by ADH1 promoter. The transformants were grown until saturation in liquid media and then spotted onto plates with or without 5-FOA. (**D**) The MMS sensitivity of *mus81* mutant alleles was examined in *sgs1Δ* background. The colonies that formed on the 5-FOA plates from (panel (C)) were inoculated in liquid media and then spotted onto plates without or with indicated amounts of MMS. The plates were incubated at 30°C for 4 days.

As shown above, the N-terminal 120 aa region of Mus81 contained two separate small regions (N21–26 and N108–114 motifs) that severely affected Rad27 binding upon their deletion. When we aligned the N-terminal regions of Mus81 from *S. cerevisiae, Schizosaccharomyces pombe* and *Homo sapiens*, the first N21–26 motif had Thr23 (T23) as the only conserved residue from yeast to humans (Figure 7A) and the second N108–114 motif did not share any noticeable conserved aa residues. The substitution of T23 into Ala (Mus81-NF120T23) did not abolish the ability of Mus81-NF120 to bind Rad27 (Figure 7B, compare lanes 14 and 17), and expression of the *mus81*_T23_ allele resulted in complementation of the growth defect of *mus81Δ* in the absence of *SGS1* (Figure 7C). Moreover, in the absence of *SGS1* the *mus81*_T23_ mutant cells showed virtually the same resistance to MMS as wild-type (Figure 7D). Since no homology was found in the second N108–114 motif, we created two mutant versions of the Mus81-NF120, namely Mus81-NF120SCE and Mus81-NF120END, by changing three consecutive Ser110, Cys111 and Glu112 (SCE_110–112_) or Glu112, Asn113 and Asp114 (END_112–114_) into Ala, respectively. We found that Mus81-NF120END was able to bind Rad27 as efficiently as Mus81-NF120 (Figure 7B, compare lanes 14 and 15), but Mus81-NF120SCE displayed a significantly reduced ability to bind Rad27 compared to wild-type (Figure 7B, compare lanes 14 and 16). Like *mus81*_T23_, the *mus81*_SCE_ mutant allele displayed the same ability to suppress the growth defect of *sgs1Δmus81Δ* and the same resistance to MMS as wild-type *MUS81* (Figure 7C and D). We then combined the two mutations (T_23_→A and SCE_110–112_→AAA) and tested whether the double mutant Mus81-NF120TSCE fragment retained the ability to bind Rad27. The result is that Mus81-NF120TSCE hardly interacted with Rad27 (Figure 7B, lane 18), indicating that Mus81-NF120TSCE lost its ability to bind Rad27. The expression of *mus81*_TSCE_ that contained the double mutations (T_23_ → A and SCE_110–112_ →AAA) in place of *MUS81* resulted in a synthetically sick phenotype when combined with *sgs1Δ* (Figure 7C). In addition, *mus81*_TSCE_ cells became hyper-sensitive to MMS, hardly growing in the presence of 0.04% MMS (Figure 7D). These results indicate that both of the two motifs, N21–26 and N108–114, were largely responsible for the physical and functional interaction between Mus81 and Rad27. The combination of T_23_ with single or double substitution in SCE_110–112_ residues, for example, *mus81*_T__23S112_, *mus81*_T__23S112C113_, *mus81*_T__23C113E114_, *mus81*_T__23S112E114_ exhibited phenotypes similar to wild-type *MUS81* (data not shown), implying that the three residues of SCE_110–112_ along with T_23_ were important collectively or in combination for the *in vivo* function of *MUS81* in the absence of *SGS1*. The more severe defect caused by the deletion of N21–26 motif than the SCE substituted mutation in N108–114 motif indicates that the N21–26 motif is necessary but not sufficient for a stable interaction between Mus81 and Rad27; it may act as a recognition site for binding Rad27, while the N108–114 motif is required to stabilize the initial interaction.

Since the levels of mutant Mus81 could affect the phenotypes observed, we replaced the strong constitutive ADH1 promoter with the endogenous one and repeated the same experiment as shown in Figures 1C, 6B and 7C. The result was that phenotypes of *mus81*_Δ120N_, *mus81*_Δ21–26_ and *mus81*_TSCE_ from the endogenous Mus81 promoter were identical to those from the ADH1 promoter (Supplementary Figure S4). This is in support of the notion that the elevated levels of enzymatic activities could not replace the outcome of a specific protein–protein interaction. In order to further confirm this, we overexpressed Rad27 in NJY1777 strain and found that the overexpression of Rad27 did not suppress the lethality of *mus81*_Δ21–26_ in the absence of *SGS1* (Supplementary Figure S5A). Moreover, it failed to suppress the MMS sensitivity of *mus81*_Δ21–26_ (Supplementary Figure S5B) or *sgs1Δmus81*_Δ21–24_ (Supplementary Figure S5C). As shown in Supplementary Figure S5D, the failure to suppress the mutant phenotypes was not due to the failure of Rad27 expression. These results above indicate that the simple enhancement of Rad27 activity by Mus81 is not sufficient for the proper functioning of the Mus81 complex *in vivo*, highlighting the importance of the physical interaction *per se* between Mus81 and Rad27.

Previously, we mapped two motifs consisting of 318–334 and 367–382 residues at the C-terminus of Rad27 within Rad27 that were required for binding and stimulation of Mus81–Mms4 activity (42). We were interested in the *in vivo* phenotype of the two deletion mutant alleles of Rad27. To this end, we used *rad27*_1*–*__366_ (C-terminal 16 aa deletion) and *rad27*_1*–*__317_ (C-terminal 65 aa deletion) that was devoid singly and doubly of the Mus81-binding motif as illustrated in Supplementary Figure S6A. The result was that both mutants exhibited MMS sensitivity similar to that of the *rad27*Δ null mutant (Supplementary Figure S6B). The MMS sensitivity of the two *rad27* mutants is not due to the failure of expression of proteins as shown in Supplementary Figure S6C. It appears that the expression levels of the two mutant proteins were much higher than the wild type, judging from the fact that the amount of wild-type Rad27 was below a detection level in this western blot. Although the mutant Rad27_1–__366_ possesses 1/4 of specific activity compared to wild type (47), the elevated levels of the mutant Rad27 could compensate its reduced enzymatic activity. Thus, this result strongly indicates that the failure of Rad27 to interact with Mus81–Mms4, not the reduced overall enzymatic activity of Rad27, is responsible for the MMS sensitivity. In addition, the sensitivity of *rad27*_1*–*__366_ to MMS (Supplementary Figure S6B) is similar to that of *mus81*_Δ120N_ (Figure 1B). These results above, taken tighter, substantiate our conclusion that the interaction between Mus81 and Rad27 has a physiological importance.

### The lethality caused by the impaired N-terminal region of Mus81 is dependent on the presence of RAD52

**Figure 8.**
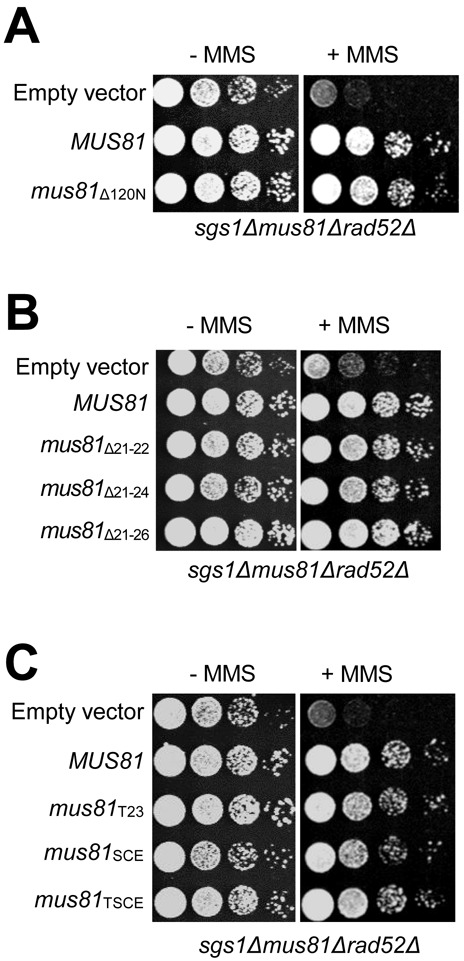
The cellular defects caused by the impaired N-terminal region of Mus81 were rescued by inactivation of Rad52. The triple deleted mutant strain MIY2343 was introduced with an empty vector or vectors containing wild-type *MUS81* and *mus81* mutant alleles that include *mus81*_Δ120N_ (**A**); *mus81*_Δ21–22_*, mus81*_Δ21–24_ and *mus81*_Δ21–26_ (**B**)*; mus81*_T23_*, mus81*_SCE_ and *mus81*_TSCE_ (**C**). The transformants were grown in liquid media until saturation and then spotted onto plates without or with 0.0008% MMS. The plates were incubated at 30°C for 4 days.

It was reported that Sgs1–Top3–Rmi1 and Mus81–Mms4 constitute parallel pathways to process toxic recombination intermediates generated by Rad52 epistasis group genes such as *RAD51* and *RAD52* (18). In addition, it was shown that the deletion of *RAD51* or *RAD52* could rescue the synthetic lethality of *sgs1Δmus81Δ* double mutant (18). This urged us to examine if the growth defects caused by the N-terminally impaired *mus81* alleles in the absence of *SGS1* could be restored by deleting *RAD52*. We found that when *RAD52* was deleted, *mus81*_Δ120N_, *mus81*_Δ21–26_ and *mus81*_TSCE_ cells, which displayed both growth defects and MMS sensitivities (Figures 1, 6 and 7, respectively), resumed to grow back as efficiently as and became as resistant to MMS as wild-type *MUS81* (Figure 8). In other words, inactivation of Rad52 not only rescued the lethality or growth defect of *sgs1Δmus81*_Δ120N_, *sgs1Δmus81*_Δ21–26_ and *sgs1Δmus81*_TSCE_ but also suppressed the MMS sensitivity of all *mus81* mutant alleles in *sgs1Δ* background (Figure 8A, B and C). These data again demonstrate that the N-terminal region of Mus81 is critical for the function of Mus81 complex in a redundant manner with Sgs1 in the HRR process.

### The *in vitro* stimulation of Mus81–Mms4 endonuclease activity by Slx1–Slx4 also requires the N-terminus of Mus81

**Figure 9.**
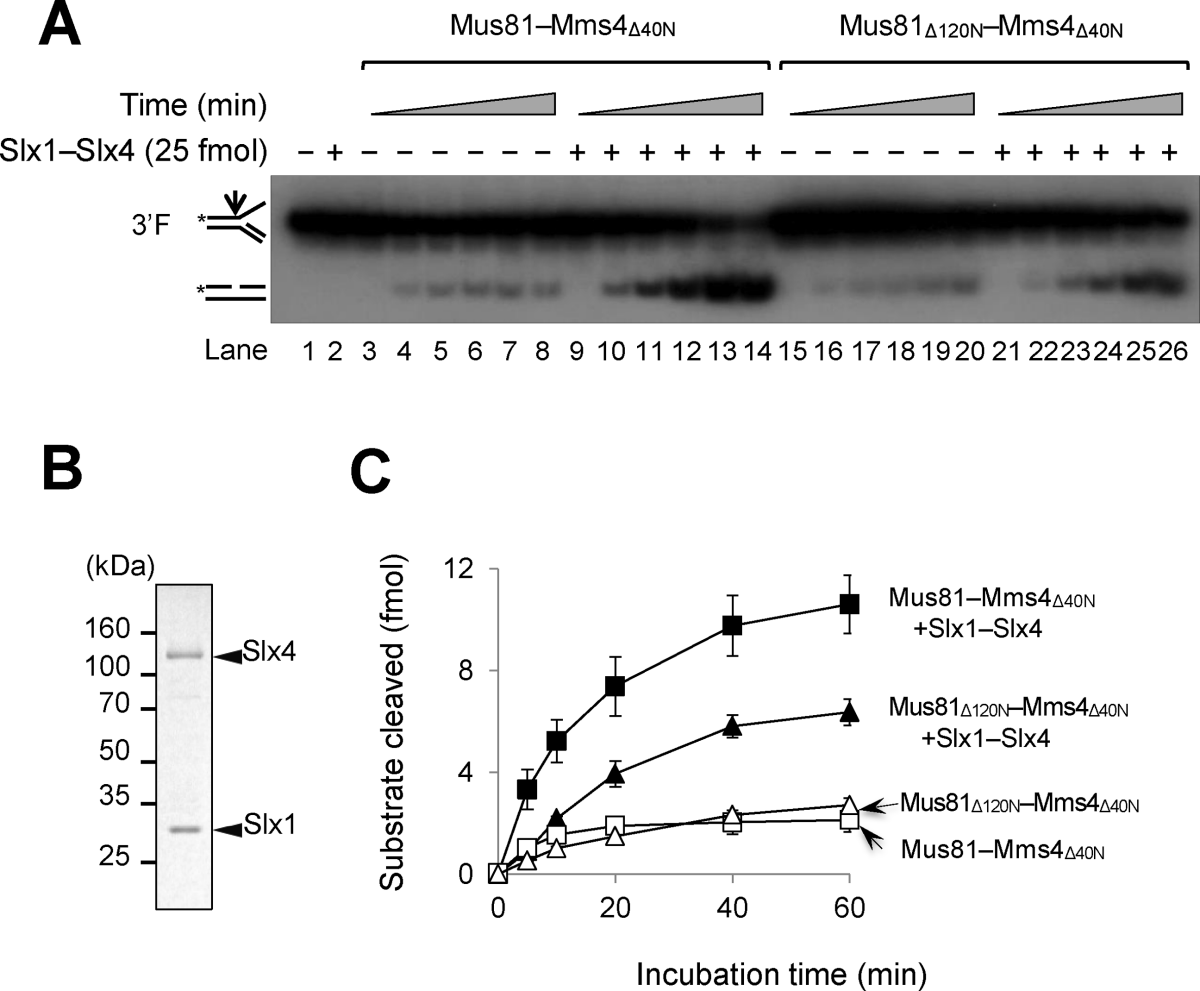
The *in vitro* stimulation of Mus81–Mms4 endonuclease activity by Slx1–Slx4 requires the N-terminus of Mus81. (**A**) A time-course assay was performed with Mus81–Mms4_Δ40N_ and Mus81_Δ120N_–Mms4_Δ40N_ (5 fmol each) in the presence or absence of Slx1–Slx4 (25 fmol). The reaction mixtures containing 15 fmol of 3′F were incubated at 30°C and aliquots of the reaction mixtures were withdrawn at 5, 10, 20, 40 and 60-min time point. (**B**) Analysis of the purified recombinant Slx1–Slx4 complex in 10% SDS-PAGE. The gel was Coomassie-blue stained. (**C**) The amounts of cleavage products obtained in (A) were plotted against incubation time. The graph with error bars was obtained from results of four independent experiments. The error bars represent the standard deviation from the mean of four independent experiments.

An alternative explanation of the synthetic lethality of *sgs1Δmus81*_Δ120N_ is that deletion of the N-terminal part of Mus81 leads to the loss of its ability to bind a factor(s) other than Rad27. One such candidate is SLX1–SLX4, another structure-specific endonuclease that can resolve 5′F, 3′F, RF and intact HJ. Recently, it was reported that human SLX1–SLX4 formed a stable complex with MUS81–EME1 in an SLX4-dependent manner during HJ resolution (48–50). Moreover, it was shown that the N-terminal 106 aa region of human MUS81 was sufficient for its interaction with SLX4 and that MUS81 mutants, which did not interact with SLX4, were defective in interstrand cross-links repair in human cells (51). Thus, it is possible that the *in vivo* defects observed with the N-terminal mutants of Mus81 in yeast could also be attributed to the failure of Mus81 to interact with Slx4. In order to examine this possibility, we investigated whether the functional interactions observed between MUS81-EME1 and SLX1–SLX4 in humans are conserved in yeasts or not, and whether those interactions, if any, are dependent on the N-terminus of Mus81. To this end, we decided to express and purified the recombinant yeast Slx1–Slx4 complex using the procedure described in the Materials and Methods section. As shown in Figure 9A, we investigated the influence of Slx1–Slx4 on the enzymatic activity of Mus81–Mms4 (Figure 9A) using the recombinant Slx1–Slx4 complex purified to near homogeneity (Figure 9B). We found that Slx1–Slx4 stimulated markedly the endonuclease activity of Mus81–Mms4_Δ40N_ and the stimulation effect reduced half when Mus81_Δ120N_–Mms4_Δ40N_ was used (Figure 9A, compare lanes 10–14 and 22–26; Figure 9C). It should be noted that Slx1–Slx4 stimulated Mus81–Mms4 as efficiently as Mus81–Mms4_Δ40N_, demonstrating that the N-terminal 40 aa region of Mms4 is not involved in the stimulation observed (data not shown). We did not detect any synergistic stimulation of Slx1–Slx4 endonuclease activity by Mus81 with 5′F and HJ substrates (data not shown), in keeping with previous findings (52). This result verifies that the N-terminus of Mus81 is important for interactions with not only Rad27 but also Slx1–Slx4, and raises the possibility that the N-terminal domain of Mus81 could contribute crucially to processing of a variety of DNA structures via its ability to interact with multiple nucleases *in vivo*.

In order to further establish that the *in vivo* defects are indeed caused by the failure of Mus81 to interact with Rad27, we decided to confirm whether the Mus81 mutants with small deletions are also as defective as Mus81_Δ120_ in being stimulated by Slx1–Slx4. In order to address this question, we examined the ability of Mus81_Δ21–26_–Mms4_Δ40N_ to be stimulated by Slx1–Slx4. In contrast to the result that Mus81–Mms4_Δ40N_ was stimulated by Rad27, but Mus81_Δ21–26_–Mms4_Δ40N_ was not (Figure 5D), Mus81_Δ21–26_–Mms4_Δ40N_ was stimulated by Slx1–Slx4 as efficiently as Mus81–Mms4_Δ40N_ (Supplementary Figure S7). Since both *mus81*_Δ120_ and *mus81*_Δ21–26_ mutant alleles shared *in vivo* defects in common, these data confirm that the *in vivo* defects are associated with the failure of Mus81 to interact with Rad27, but not with Slx1–Slx4. It should be noted that Slx1 failed to coimmunoprecipitate with Mus81 (data not shown) in contrast to Rad27 with Mus81, indicating that the interaction of Mus81, if any, with Slx1 is much weaker than that of Mus81 with Rad27. This is in keeping with the previous report that no interaction was detected between Slx4 and Mus81–Mms4 when immunoprecipitation was used (52).

## DISCUSSION

In order to establish the physiological significance of the functional interactions between Mus81 and Rad27, we attempted to map the specific region in Mus81 responsible for the protein–protein interaction with Rad27 and found that the N-terminal region of Mus81 was dispensable for its catalytic activity, but critical for its *in vivo* function. This finding is congruent with the fact that the C-terminus of Mus81 is essential to form a complex with Mms4 (24) and with the previous finding obtained from studies with humans (53). Results from recent studies showed that the N-terminal 244 aa deletion of human MUS81 caused significant reduction in endonuclease activities of both MUS81–EME1 and MUS81–EME2 complexes due to the loss of the highly conserved winged helix domain (54). However, the deletion of 120 aa residues from the N-terminus of Mus81 and other mutant derivatives used in this study did not lead to reduction in endonuclease activity of the Mus81 complexes because a winged helix domain of yeast Mus81 is located from aa residues 129 to 244 at its N-terminus.

Although the Mus81_Δ120N_ subunit alone failed to bind Rad27 (Figure 1A), the Mus81_Δ120N_–Mms4_Δ40N_ complex was able to stimulate Rad27 activity as efficiently as Mus81–Mms4 or Mus81–Mms4_Δ40N_ (Figure 3A and B). We found that purified recombinant Mms4 alone was able to stimulate Rad27 activity (data not shown), implying that the stimulation of Rad27 activity by the Mus81 complexes is also mediated through the interaction between Mms4 and Rad27. Although we failed to detect stable interaction between Mms4 and Rad27 using coimmunoprecipitation, they were reported to have direct physical interaction according to Saccharomyces Genome Database. Thus, our *in vivo* and *in vitro* findings indicate that the impaired interaction between Mus81 and Rad27, but not between Mms4 and Rad27, could be an underlying cause of cellular defects we observed. It would be interesting if we could determine the domain of Mms4 responsible to interact with Rad27 and analyze a cellular defect caused by the impaired interaction between Mms4 and Rad27. It would be particularly informative if we could isolate *mus81mms4* double mutants that have lost entirely the ability to interact with Rad27 and define their cellular defects. We believe that these double mutants would be more severely defective than the *mus81* mutant alleles used in this study.

In normally growing mitotic cells, it appears that the HR intermediates are resolved preferentially via a process called ‘dissolution’ by the Sgs1–Top3–Rmi1 complex since this process would produce non-crossover products only, which minimizes alteration of genetic materials. When cells are treated with DNA damaging agents, they may interfere with normal progression of RFs or induce excess DSBs. Under these circumstances, the joint action of Mus81–Mms4 and Rad27 could contribute critically to cell viability by rapidly processing the excess levels of DNA damages, although at the risk of crossover events. The Sgs1–Top3–Rmi1 pathway is a preferred choice to mitotic cells in a sense that it does not form crossover products. Under special circumstances, however, when joint recombination intermediates are formed between two identical sister chromatids, the way by which the recombination intermediates are resolved does not affect the integrity of resolution products. This is because the recombination products formed via crossover and non-crossover events are identical. This idea is in keeping with our finding that Rad27, which is to process Okazaki fragments during lagging strand synthesis, is associated with Mus81–Mms4.

We believe that the alternative Sgs1–Top3–Rmi1 pathway is not sufficient or efficient enough to deal with the excess levels of DNA damages from either endogenous or exogenous sources for the following two reasons. (i) From a mechanistic viewpoint, Sgs1–Top3–Rmi1 would not be involved in the initial stage of restoration of stalled RFs or formation of double HJ from DSBs, only being able to use topologically linked intermediates as its substrate that are formed by action of other enzymes. (ii) From a kinetic viewpoint, it is likely that the dissolution of double HJs by Sgs1–Top3–Rmi1 would be more complex in its action cycle (binding–unwinding–relaxation) and, hence, might be intrinsically slower than the simple catalytic action of Mus81–Mms4 enzyme. Given that Sgs1–Top3–Rmi1 has a mechanistic and kinetic limitation in its action, the Mus81–Mms4 endonuclease activity could play a major role in the processing of the excess toxic recombination intermediates.

We also found that the deletion of the N21–26 motif resulted in poor binding of Mus81-NF120 to Rad27 and this motif is critical for the Rad27-mediated stimulation of Mus81 activity (Figure 5A and D, respectively). Most importantly, the *mus81*_Δ21–26_ mutant allele did not support cell growth in the absence of *SGS1* (Figure 6B), indicating that the N21–26 motif is essential for the *in vivo* function of the Mus81–Mms4 complex. Although the substitution of S_110_C_111_E_112_ into AAA within the N-terminus of Mus81 resulted in a significant reduction in Rad27 binding, it did not give rise to any detectable cellular defect (Figure 7C and D). The *mus81*_SCE_ mutant displayed synergistic defects only when combined with a T_23_→A substitution; Mus81-NF120TSCE was almost unable to bind Rad27 (Figure 7B); *mus81*_TSCE_ cells were synthetically sick (Figure 7C). It should be noted that the substitution of T_23_ might not completely alter the structure of N21–26, preserving the residual function of Mus81 and allowing the mutant cells to grow to a limited extent in *sgs1Δ* background. Based upon these observations, we propose that the bipartite N21–26 and N108–114 motifs in Mus81 contribute to the specific and stable binding of Mus81 to Rad27; the former is required for specificity, while the latter for binding stability. With an intact N21–26 motif only, the interaction of Mus81 with Rad27 might be weak, but productive enough to support its normal function *in vivo*. In contrast, intact N108–114 motif alone leads to nonspecific, and thus most likely nonproductive interaction of Mus81 with Rad27, resulting in the cellular defects observed.

Our findings (i) that *mus81*_Δ120N_ and *mus81*_Δ21–26_, both of which are catalytically active, were as defective as *mus81Δ in vivo* and (ii) that the synthetic lethality of both *sgs1Δmus81*_Δ120N_ and *sgs1Δmus81*_Δ21–26_ was rescued by deletion of *RAD52* highlight the physiological importance of the N-terminal region of Mus81, placing Rad27 as an important functional partner in removing toxic recombination intermediates. The joint role of Rad27 and Mus81–Mms4 could be of particular use because it could allow rapid removal of branched ssDNAs interconvertible between 5′ and 3′ flaps via double-flap intermediates; the 5′ flap can be converted into a 3′ flap or vice versa, in a manner similar to that seen in HJ migration.

Mus81 contains tandem helix-hairpin-helix (HhH) motifs that are positioned at either end of the protein; the N-terminal HhH motif is located from 51 to 70 residues while the C-terminal HhH motif is positioned from 554 to 591 residues (16). The HhH motifs were reported to play a role in DNA binding and substrate recognition; in the predicted three-dimensional structure of Mus81, these two motifs should be close to each other (3). The N-terminal HhH motif was dispensable for the physical interaction of Mus81 with Mms4 since the C-terminal fragment of Mus81 from 527 to 632 residues containing the C-terminal HhH motif was sufficient for complex formation with Mms4 (24). Here, we found the two small motifs, namely N21–26 and N108–114, flanking the N-terminal HhH motif were crucial for physical and functional interaction between Mus81 and Rad27. These results suggest that while the C-terminal HhH motif is responsible for binding a non-catalytic partner Mms4, the N-terminal HhH motif and flanking sequences are in charge of binding an additional interacting partner such as Rad27. Based upon this and our data above, we propose that the N-terminus of Mus81 interacts with the C-terminus of Rad27 and this binding is important for the functional interaction between two enzymes and for the cellular function of Mus81.

Recently, it was shown that SLX1–SLX4 functions together with MUS81–EME1 in resolving recombination intermediates including HJ in humans (48–50). We found that yeast Slx1–Slx4 significantly stimulated Mus81–Mms4 with 3′F substrate in a manner dependent on the N-terminal region of Mus81 (Figure 9). In contrast to this finding, several previous reports suggest that yeast Slx1–Slx4 did not work together with Mus81 during HRR. Although deletion of *RAD52* suppressed all *mus81* mutant alleles in the *sgs1* null background, mutations of *RAD52* failed to rescue the lethality of *sgs1Δslx1Δ* or *sgs1Δslx4Δ*, implying that Slx1–Slx4 is not involved in Rad52-dependent recombination (46). Besides, *slx1Δ* did not exhibit the sensitivity to MMS and HU in either budding or fission yeast whereas *sgs1Δ* and *mus81Δ* mutants are highly sensitive to those DNA damaging agents, suggesting that Mus81 and Slx1 function in separate pathways (9,55,56). Recently, however, Slx4–Dpb11 was reported to form a multiprotein complex with Mus81–Mms4 (57). The formation of this complex depends on Cdk1-dependent phosphorylation of Slx4 and Cdc5-dependent phosphorylation of Mms4, indicating the function of Slx4–Dpb11 in activation of Mus81–Mms4 activity in the resolution of jointed-molecule structures. It was shown that the phosphorylation of Mms4 is critical for the function of Mus81 at G2/M, activating the basal activity of Mus81 and facilitating its association with interacting partner(s). However, the phosphorylation of Mms4 may not be important for the Mus81 function in association with Rad27, since processing endogenous toxic recombination intermediates arising from DNA replication errors takes place during S phase. Considering that Rad27 plays diverse roles in a number of DNA transactions, it is likely that *in vivo* Rad27 is more abundant than Slx1. Thus, Rad27 binds Mus81 more favorably than Slx1 when they have to compete for the same binding domain of Mus81. We believe that Mus81 could associate with Slx1 temporarily and in a regulated manner. For example, a modification such as phosphorylation may be required for Slx1 to productively associate with Mus81 for a specific cellular function as reported with human MUS81–EME1 and SLX1–SLX4 (50) or yeast Mus81–Mms4 and Slx4–Dbp11 (57); both EME1 and SLX4 in humans (Mms4 and Slx4 in yeasts) undergo phosphorylation during the G2/M phase, resulting in complex formation.

In summary, the interaction of Mus81–Mms4 with multiple nucleases including Rad27 and Slx1–Slx4 could allow Mus81–Mms4 to form a multi-functional nuclease complex that can act more efficiently in restoring stalled RF or resolving a wide range of aberrant DNA structures including toxic recombination intermediates. In addition, it would also provide an excellent point of regulation of their activities, for example, via covalent modification such as phosphorylation. Further analysis of the functional interactions between Mus81–Mms4 and Rad27/Slx1–Slx4 will shed light into the precise physiological functions of these structure-resolving nucleases and their regulation during the cell cycle.

## SUPPLEMENTARY DATA

Supplementary Data are available at NAR Online.

SUPPLEMENTARY DATA
