# Supplementary material for: A physiological significance of the functional interaction between Mus81 and Rad27 in homologous recombination repair
Source: Nucleic Acids Res. 2015 Jan 27;43(3):1684–99. doi: 10.1093/nar/gkv025 (PMC4330386; doi:10.1093/nar/gkv025)
Supplement: SUPPLEMENTARY DATA [file supp_43_3_1684__index.html]

A physiological significance of the functional interaction between Mus81 and Rad27 in homologous recombination repair — SUPPLEMENTARY DATA 

# A physiological significance of the functional interaction between Mus81 and Rad27 in homologous recombination repair

## SUPPLEMENTARY DATA

**Files in this Data Supplement:**

- Supplementary Figures
