# Supplementary material for: A physiological significance of the functional interaction between Mus81 and Rad27 in homologous recombination repair
Source: Nucleic Acids Res. 2015 Jan 27;43(3):1684–99. doi: 10.1093/nar/gkv025 (PMC4330386; doi:10.1093/nar/gkv025)
Supplement: SUPPLEMENTARY DATA [file supp_gkv025_150121-Supplement-FiguresNAR-03242-D-2014.pptx]

## Slide 1
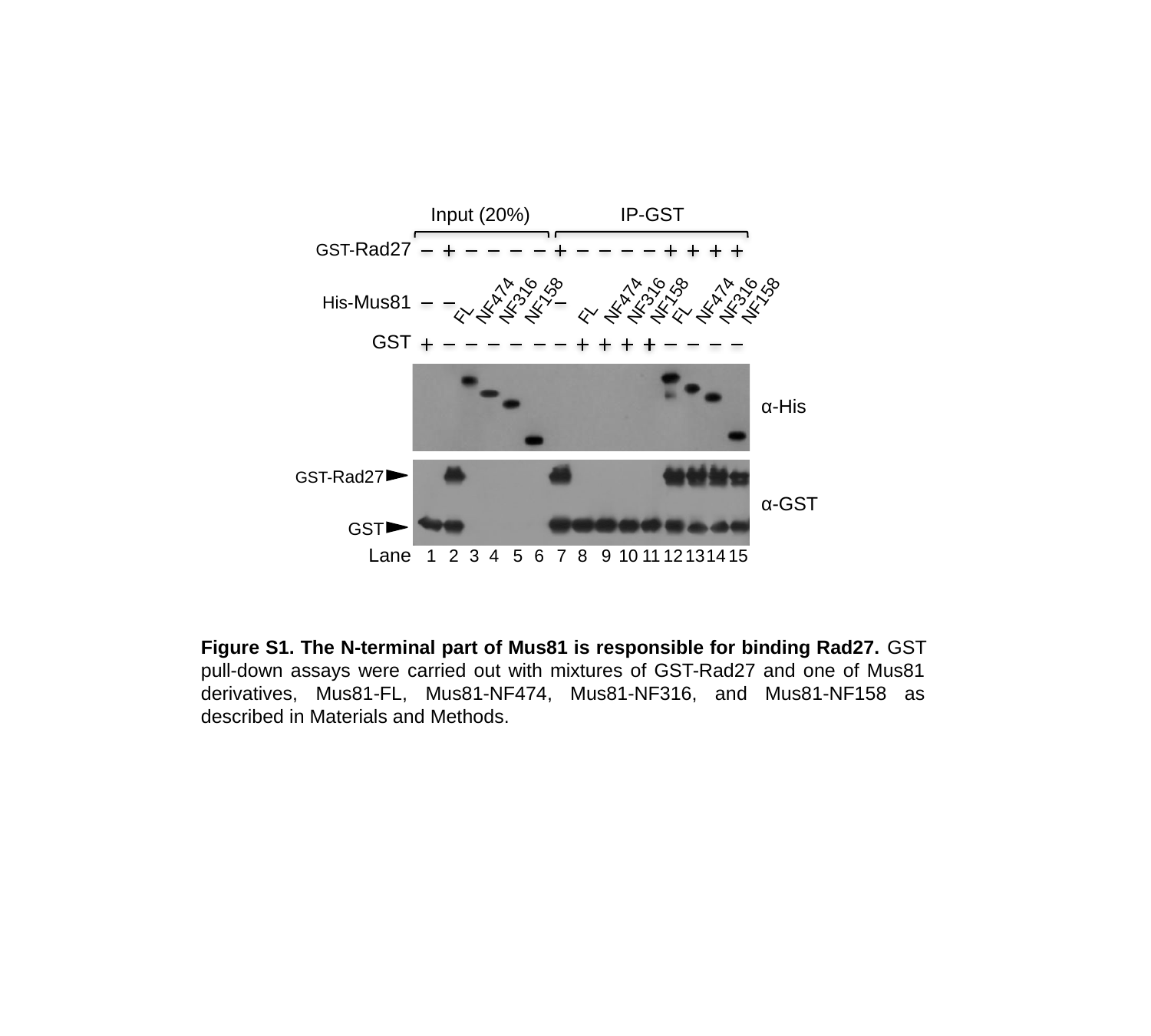

Input (20%)
IP-GST
GST-Rad27
NF316
NF474
NF474
NF316
NF158
NF474
NF316
NF158
NF158
His-Mus81
FL
FL
FL
GST
α-His
GST-Rad27
α-GST
GST
Lane
1
2
3
4
5
6
7
8
9
10
11
12
13
14
15
Figure S1. The N-terminal part of Mus81 is responsible for binding Rad27. GST pull-down assays were carried out with mixtures of GST-Rad27 and one of Mus81 derivatives, Mus81-FL, Mus81-NF474, Mus81-NF316, and Mus81-NF158 as described in Materials and Methods.

## Slide 2
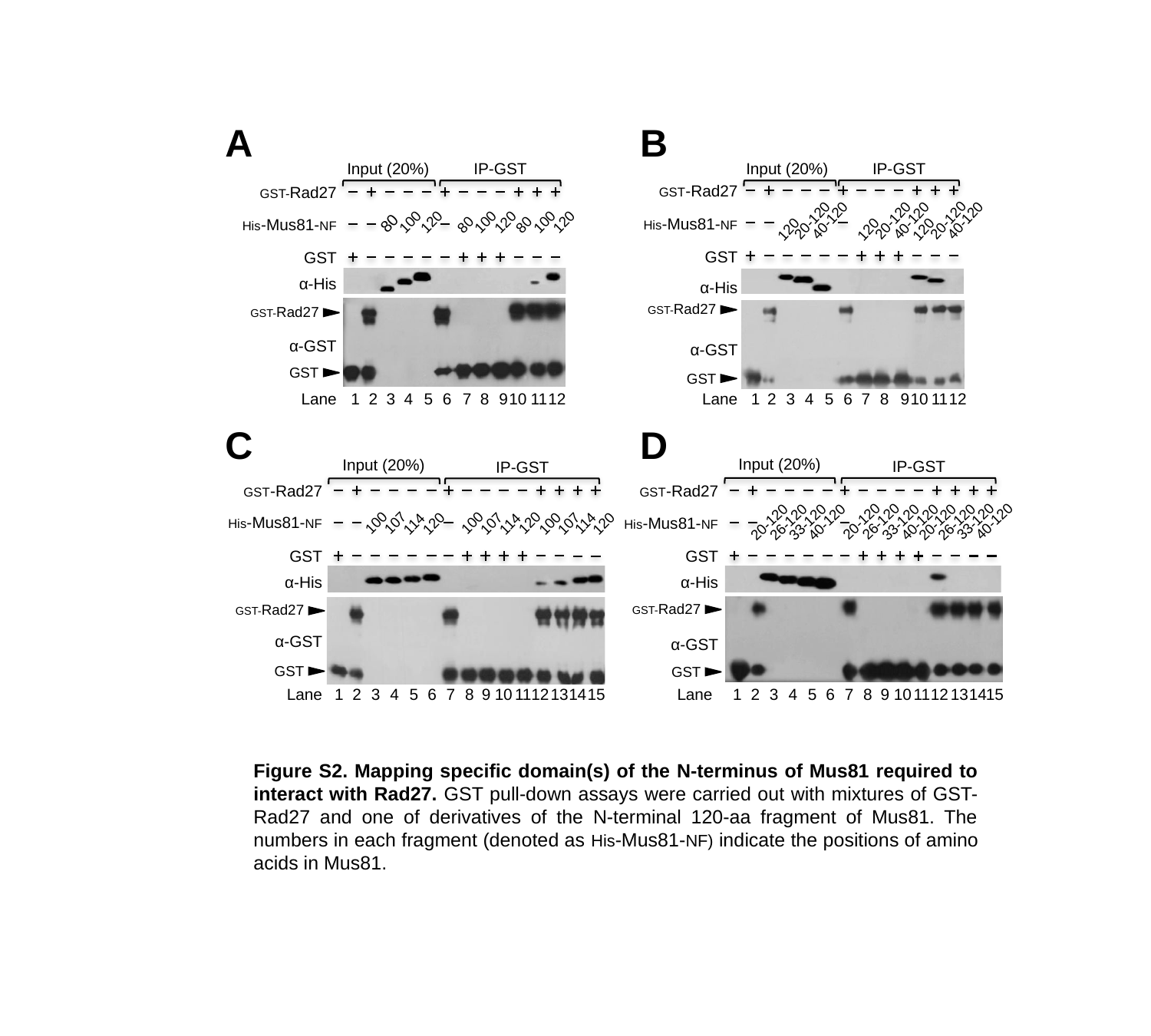

A
B
IP-GST
Input (20%)
Input (20%)
IP-GST
GST-Rad27
GST-Rad27
40-120
120
20-120
40-120
20-120
20-120
40-120
100
120
100
100
120
80
His-Mus81-NF
His-Mus81-NF
80
80
120
120
120
GST
GST
α-His
α-His
GST-Rad27
GST-Rad27
α-GST
α-GST
GST
GST
Lane
1
2
3
4
5
6
7
8
9
10
11
12
Lane
1
2
3
4
5
6
7
8
9
10
11
12
D
C
Input (20%)
Input (20%)
IP-GST
IP-GST
GST-Rad27
GST-Rad27
20-120
33-120
40-120
33-120
40-120
20-120
26-120
107
100
26-120
26-120
120
114
33-120
100
20-120
120
114
107
114
100
40-120
His-Mus81-NF
His-Mus81-NF
120
107
GST
GST
α-His
α-His
GST-Rad27
GST-Rad27
α-GST
α-GST
GST
GST
Lane
1
2
3
4
5
6
7
8
10
9
11
12
13
14
15
Lane
1
2
3
4
5
6
7
8
10
9
11
12
13
14
15
Figure S2. Mapping specific domain(s) of the N-terminus of Mus81 required to interact with Rad27. GST pull-down assays were carried out with mixtures of GST-Rad27 and one of derivatives of the N-terminal 120-aa fragment of Mus81. The numbers in each fragment (denoted as His-Mus81-NF) indicate the positions of amino acids in Mus81.

## Slide 3
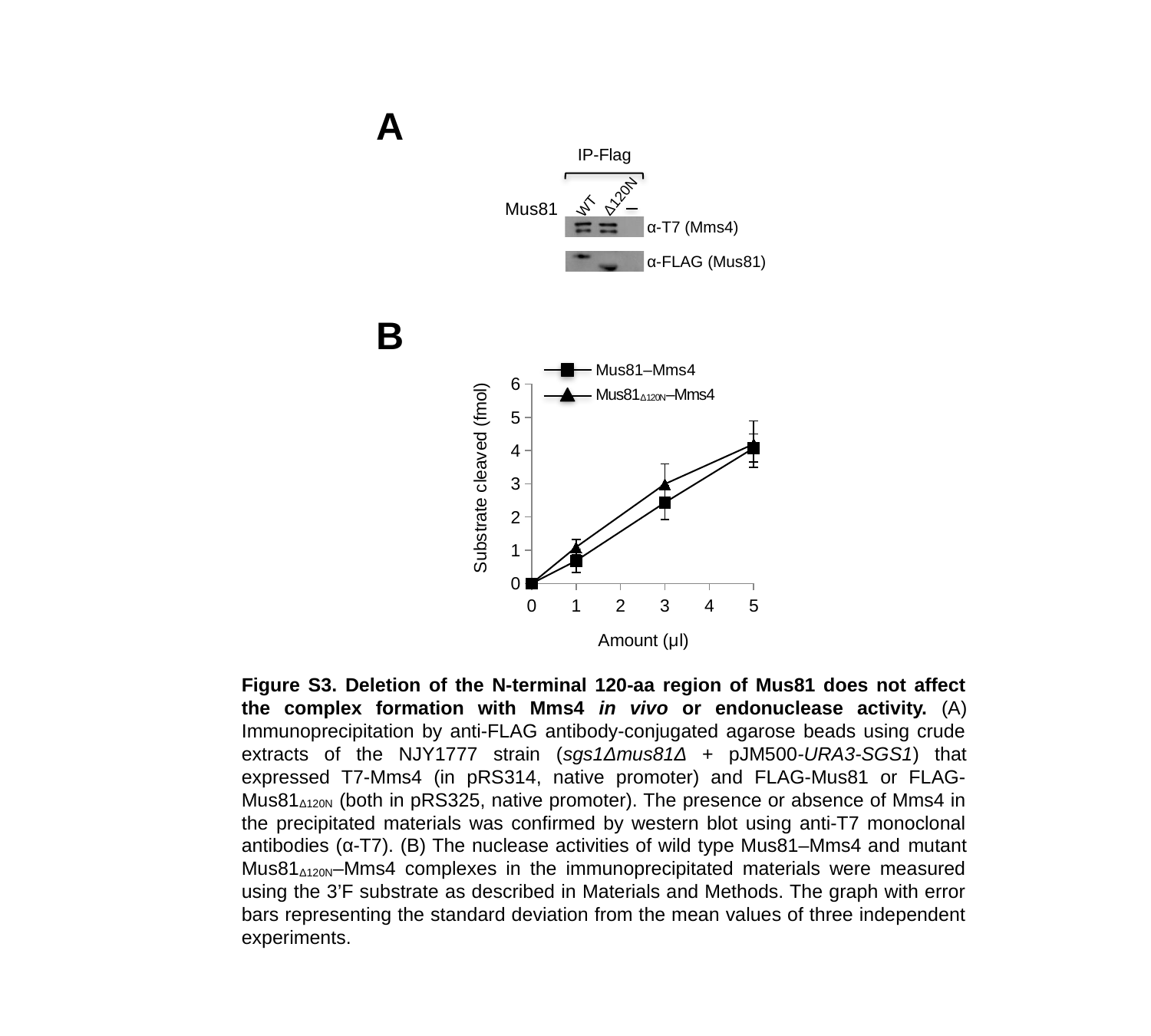

A
IP-Flag
Δ120N
WT
Mus81
α-T7 (Mms4)
α-FLAG (Mus81)
B
### Chart
| Category | | |
|---|---|---|Mus81–Mms4
Mus81Δ120N–Mms4
Figure S3. Deletion of the N-terminal 120-aa region of Mus81 does not affect the complex formation with Mms4 in vivo or endonuclease activity. (A) Immunoprecipitation by anti-FLAG antibody-conjugated agarose beads using crude extracts of the NJY1777 strain (sgs1Δmus81Δ + pJM500-URA3-SGS1) that expressed T7-Mms4 (in pRS314, native promoter) and FLAG-Mus81 or FLAG-Mus81Δ120N (both in pRS325, native promoter). The presence or absence of Mms4 in the precipitated materials was confirmed by western blot using anti-T7 monoclonal antibodies (α-T7). (B) The nuclease activities of wild type Mus81–Mms4 and mutant Mus81Δ120N–Mms4 complexes in the immunoprecipitated materials were measured using the 3’F substrate as described in Materials and Methods. The graph with error bars representing the standard deviation from the mean values of three independent experiments.

## Slide 4
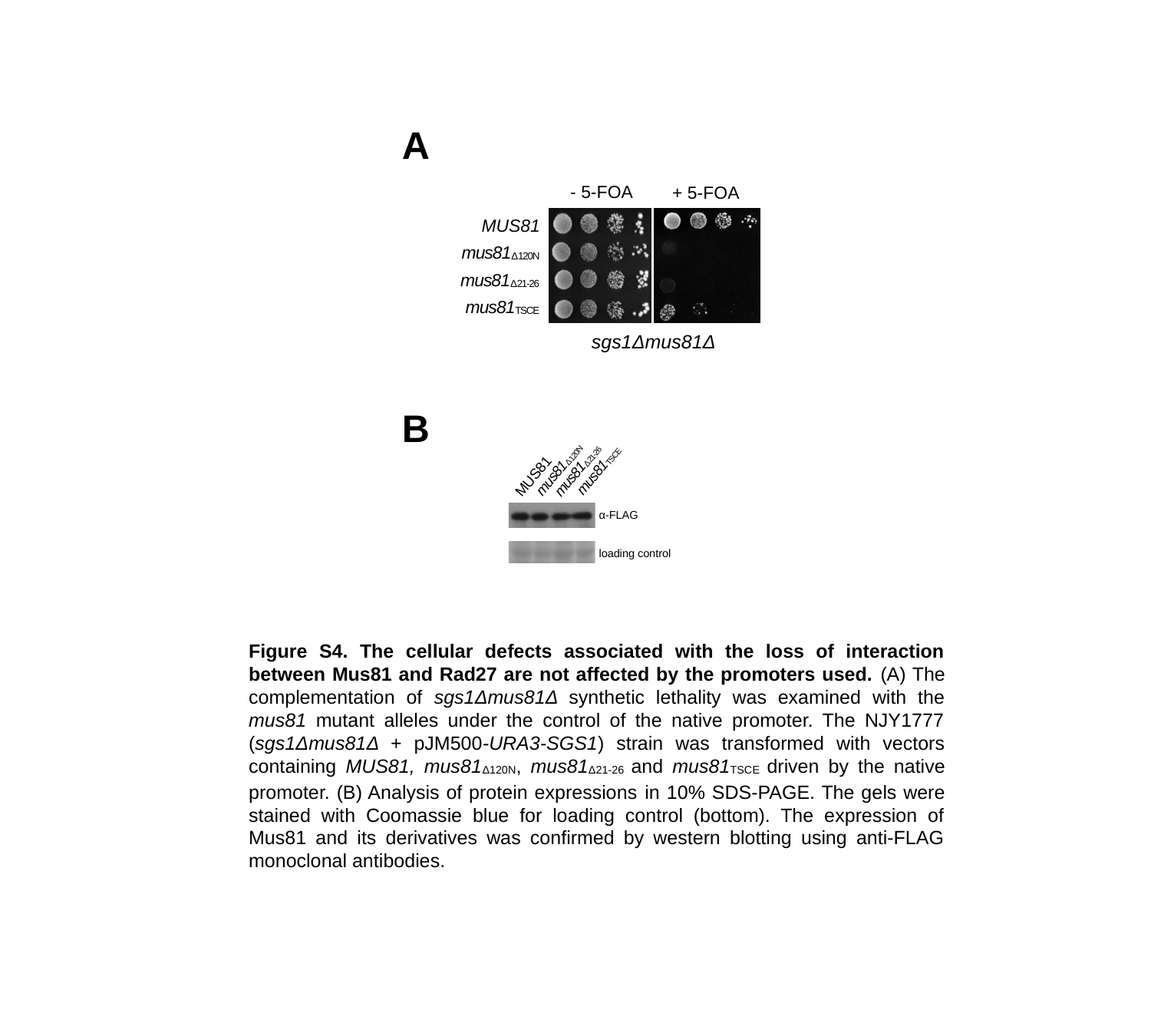

A
- 5-FOA
+ 5-FOA
MUS81
mus81Δ120N
mus81Δ21-26
mus81TSCE
sgs1Δmus81Δ
B
MUS81
mus81Δ21-26
mus81Δ120N
mus81TSCE
α-FLAG
loading control
Figure S4. The cellular defects associated with the loss of interaction between Mus81 and Rad27 are not affected by the promoters used. (A) The complementation of sgs1Δmus81Δ synthetic lethality was examined with the mus81 mutant alleles under the control of the native promoter. The NJY1777 (sgs1Δmus81Δ + pJM500-URA3-SGS1) strain was transformed with vectors containing MUS81, mus81Δ120N, mus81Δ21-26 and mus81TSCE driven by the native promoter. (B) Analysis of protein expressions in 10% SDS-PAGE. The gels were stained with Coomassie blue for loading control (bottom). The expression of Mus81 and its derivatives was confirmed by western blotting using anti-FLAG monoclonal antibodies.

## Slide 5
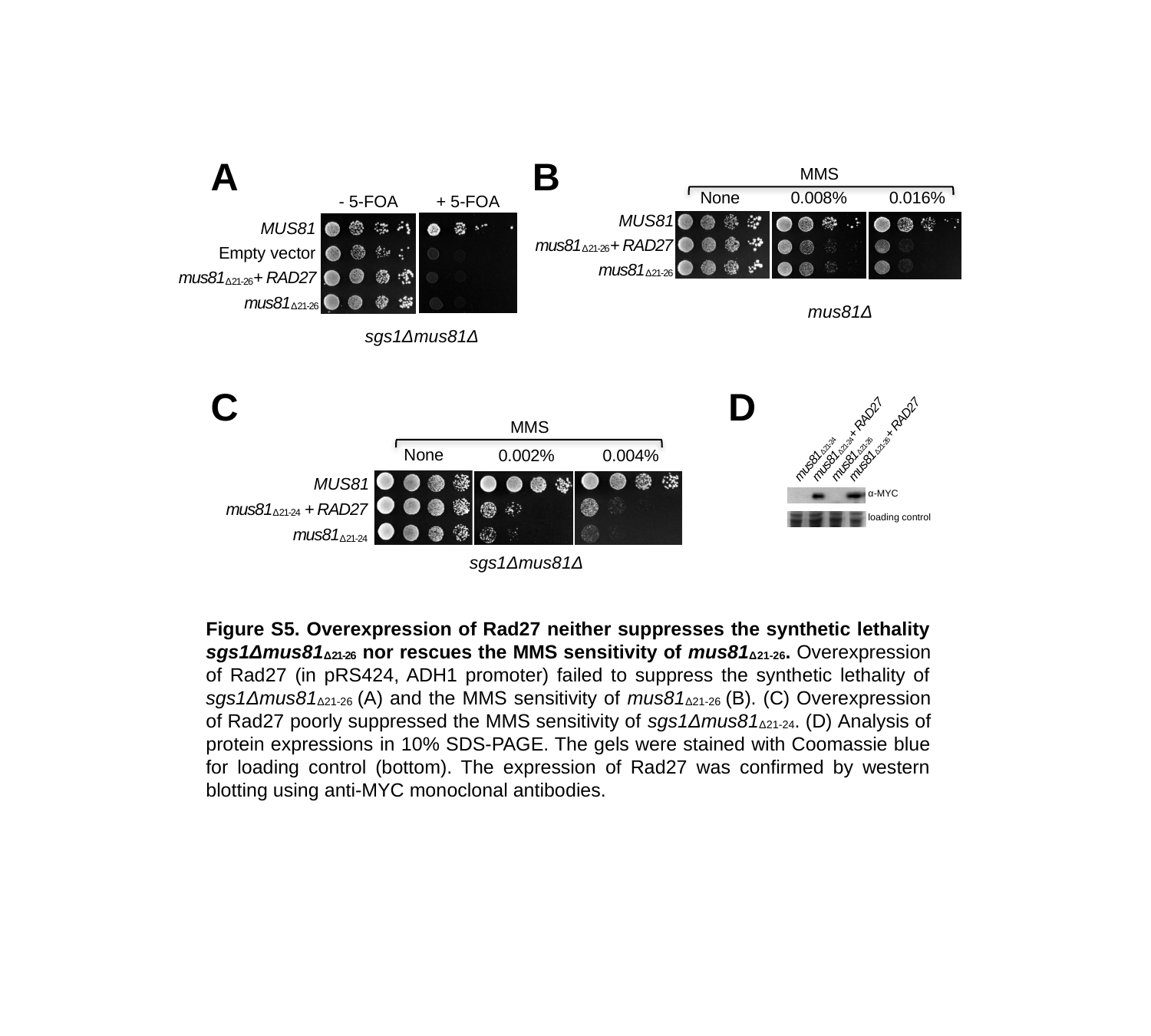

A
B
MMS
None
0.008%
0.016%
- 5-FOA
+ 5-FOA
MUS81
MUS81
mus81Δ21-26+ RAD27
Empty vector
mus81Δ21-26
mus81Δ21-26+ RAD27
mus81Δ21-26
mus81Δ
sgs1Δmus81Δ
C
D
mus81Δ21-24+ RAD27
mus81Δ21-26+ RAD27
MMS
mus81Δ21-24
mus81Δ21-26
None
0.002%
0.004%
MUS81
α-MYC
mus81Δ21-24 + RAD27
loading control
mus81Δ21-24
sgs1Δmus81Δ
Figure S5. Overexpression of Rad27 neither suppresses the synthetic lethality sgs1Δmus81Δ21-26 nor rescues the MMS sensitivity of mus81Δ21-26. Overexpression of Rad27 (in pRS424, ADH1 promoter) failed to suppress the synthetic lethality of sgs1Δmus81Δ21-26 (A) and the MMS sensitivity of mus81Δ21-26 (B). (C) Overexpression of Rad27 poorly suppressed the MMS sensitivity of sgs1Δmus81Δ21-24. (D) Analysis of protein expressions in 10% SDS-PAGE. The gels were stained with Coomassie blue for loading control (bottom). The expression of Rad27 was confirmed by western blotting using anti-MYC monoclonal antibodies.

## Slide 6
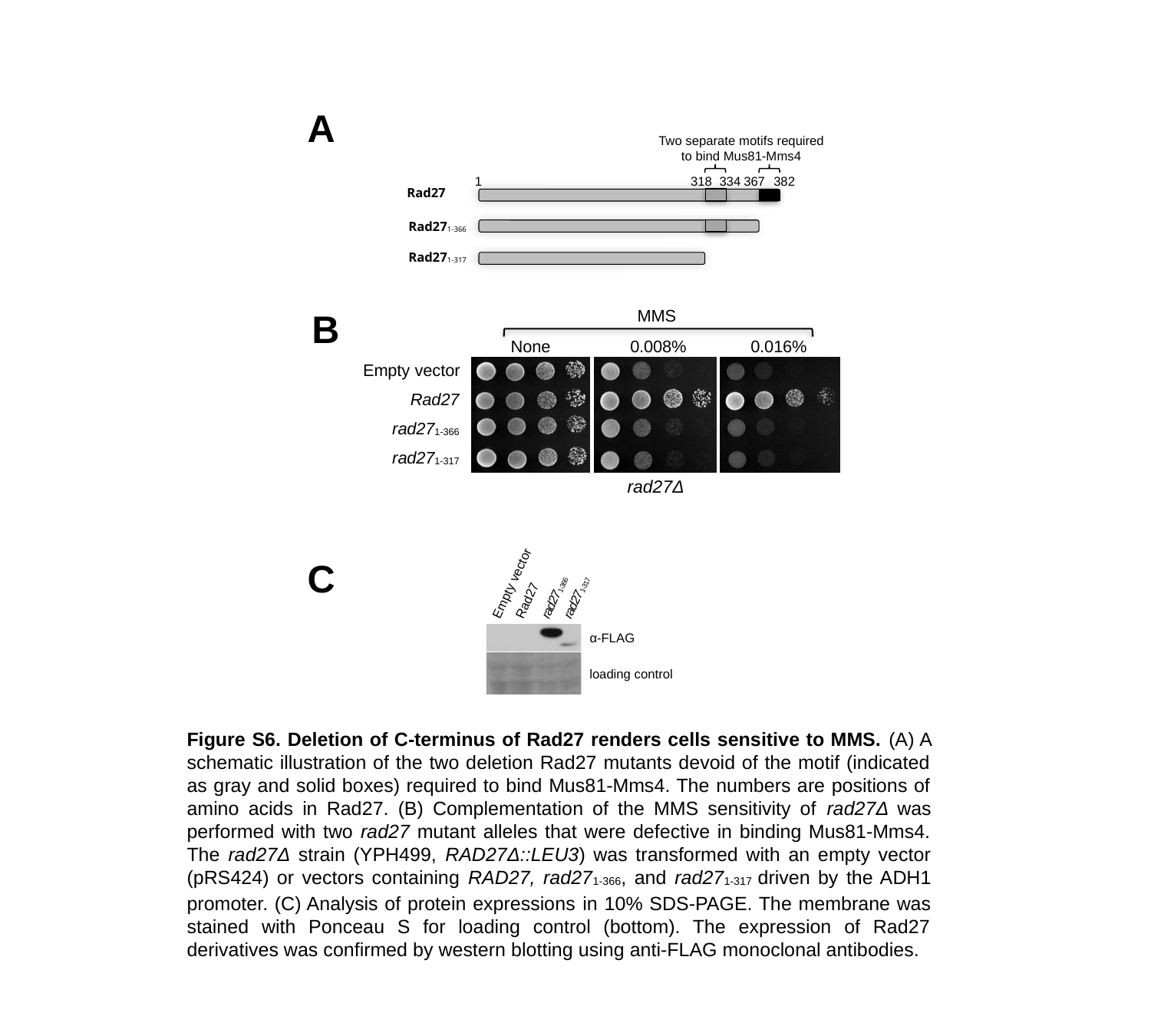

A
Two separate motifs required to bind Mus81-Mms4
1
318
334
367
382
Rad27
Rad271-366
Rad271-317
B
MMS
None
0.008%
0.016%
Empty vector
Rad27
rad271-366
rad271-317
rad27Δ
C
Empty vector
Rad27
rad271-366
rad271-317
α-FLAG
loading control
Figure S6. Deletion of C-terminus of Rad27 renders cells sensitive to MMS. (A) A schematic illustration of the two deletion Rad27 mutants devoid of the motif (indicated as gray and solid boxes) required to bind Mus81-Mms4. The numbers are positions of amino acids in Rad27. (B) Complementation of the MMS sensitivity of rad27Δ was performed with two rad27 mutant alleles that were defective in binding Mus81-Mms4. The rad27Δ strain (YPH499, RAD27Δ::LEU3) was transformed with an empty vector (pRS424) or vectors containing RAD27, rad271-366, and rad271-317 driven by the ADH1 promoter. (C) Analysis of protein expressions in 10% SDS-PAGE. The membrane was stained with Ponceau S for loading control (bottom). The expression of Rad27 derivatives was confirmed by western blotting using anti-FLAG monoclonal antibodies.

## Slide 7
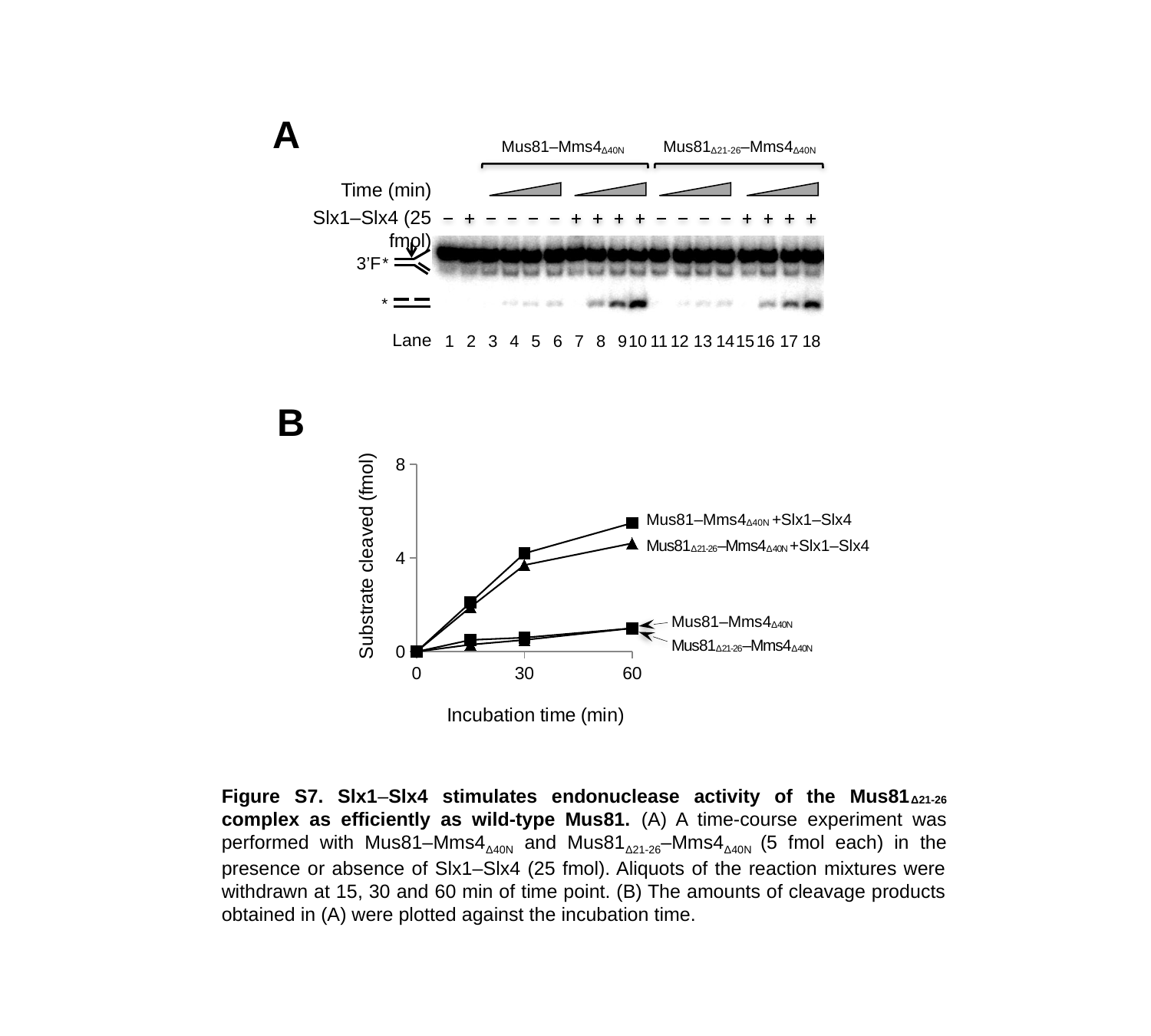

A
Mus81–Mms4Δ40N
Mus81Δ21-26–Mms4Δ40N
Time (min)
Slx1–Slx4 (25 fmol)
*
3’F
*
Lane
1
2
3
4
5
6
7
8
9
10
11
12
13
14
15
16
17
18
B
### Chart
| Category | | | | |
|---|---|---|---|---|Mus81–Mms4Δ40N +Slx1–Slx4
Mus81Δ21-26–Mms4Δ40N +Slx1–Slx4
Mus81–Mms4Δ40N
Mus81Δ21-26–Mms4Δ40N
Figure S7. Slx1–Slx4 stimulates endonuclease activity of the Mus81Δ21-26 complex as efficiently as wild-type Mus81. (A) A time-course experiment was performed with Mus81–Mms4Δ40N and Mus81Δ21-26–Mms4Δ40N (5 fmol each) in the presence or absence of Slx1–Slx4 (25 fmol). Aliquots of the reaction mixtures were withdrawn at 15, 30 and 60 min of time point. (B) The amounts of cleavage products obtained in (A) were plotted against the incubation time.
